# Separation and Detection of Charged Unilamellar Vesicles in Vacuum by a Frequency-Controlled Quadrupole Mass Sensor

**DOI:** 10.1021/acs.analchem.4c05730

**Published:** 2025-04-22

**Authors:** Anatolii Spesyvyi, Marek Cebecauer, Ján Žabka, Agnieszka Olżyńska, Michaela Malečková, Zuzana Johanovská, Miroslav Polášek, Ales Charvat, Bernd Abel

**Affiliations:** † J. Heyrovský Institute of Physical Chemistry of the Czech Academy of Sciences, Prague 18223, Czechia; ‡ Faculty of Mathematics and Physics, Charles University, Prague 12116, Czechia; § Institute of Chemical Technology, Leipzig University, Leipzig 04103, Germany; ∥ Leibniz Institute of Surface Engineering, Leipzig 04318, Germany

## Abstract

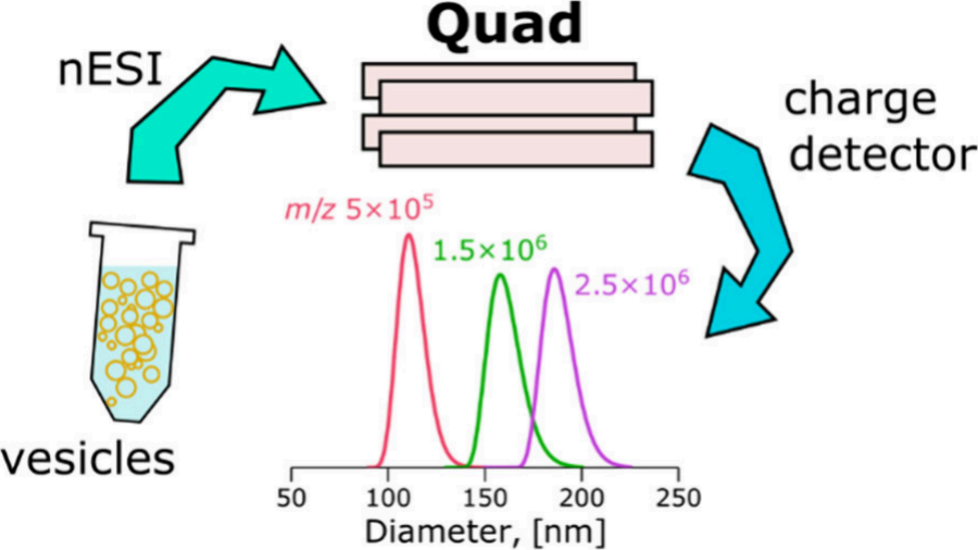

Extracellular vesicles (EVs) are membranous particles released by cells and are considered to be promising sources of biomarkers for various diseases. Mass spectrometry (MS) analysis of EVs requires a sample of purified and detergent-lysed EVs. Purification of EVs is laborious, based on size, density, or surface nature, and requires large amounts of the source material (e.g., blood, spinal fluid). We have employed synthetically produced large unilamellar lipid vesicles (LUVs) as analogs of EVs to demonstrate an alternative approach to vesicle separation for subsequent mass spectrometry analysis of their composition. Mass-to-charge ratio *m*/*z* separation by frequency-controlled quadrupole was employed to filter narrow-size distributions of LUVs from a water sample. Lipid vesicles were positively charged with nanoelectrospray and transferred into a vacuum using two wide *m*/*z*-range frequency-controlled quadrupoles. The *m*/*z*, charges, and masses of individual vesicles were obtained by the nondestructive single-pass charge detector. The resolving mode of the second quadrupole with *m*/*z* RSD < 10% allowed to separate size selected distributions of vesicles with modal diameters of 88, 112, 130, 162, and 190 nm at corresponding quadrupole *m*/*z* settings of 2.5 × 10^5^, 5 × 10^5^, 8 × 10^5^, 1.5 × 10^6^, and 2.5 × 10^6^, respectively with a rate of 20–100 counts per minute. The distributions of bioparticles with masses between 10^8^ and 10^10^ Da were separated from human blood serum in the pilot experiment. The presented approach for lipid vesicle separation encourages the development of new techniques for the direct mass-spectrometric analysis of biomarkers in MS-separated EVs in a vacuum.

Extracellular vesicles (EVs) are membranous particles^1^ released by diverse cell types. EVs participate in intercellular transport and communication.^2^ Their cargo may include proteins, nucleic acids, lipids, and cell metabolites, which can be valuable biomarkers of human diseases or metabolic changes.^3,4^ Mass spectrometry (MS) analysis of the EVs’ cargo composition requires isolated and lysed (membrane disintegration) vesicles.^5^ The EVs’ isolation from biological samples (e.g., blood plasma, urine) is laborious and time-consuming and includes ultracentrifugation, filtering, size-exclusion chromatography, or affinity-based purification on functionalized beads.^6^ Established separation techniques usually require larger amounts of material, which can be limited in samples from children or in the case of cerebrospinal fluid. The size of isolated vesicles is then characterized by dynamic light scattering, nanoparticle tracking analysis, flow cytometry, or transmission electron microscopy, which further reduces the yield of EV separation.^7^ The size of the analyzed EVs is a critical parameter. There is a large variety of EVs produced by cells, and those within a smaller range (50–300 nm in diameter) are considered more informative about the cell state or the local environment than larger EVs.^8^ Large unilamellar vesicles (LUVs), i.e., synthetic liposomes of size 100–1000 nm with a single outer lipid bilayer and enclosed aqueous core, are an established model system of EVs, particularly because of size and shape similarity and the fact they mimic mechanical and structural properties of the membrane and its functionalization with proteins.^9−11^ 1-palmitoyl-2-oleoyl-glycero-3-phosphocholine used for synthetic vesicle preparation for this study is a common lipid mimicking cellular membrane in model systems.^12^ The characterization of charged liposomes based on their gas-phase mobility was introduced as a nanoelectrospray gas-phase electrophoretic mobility molecular analyzer (nES GEMMA) and was applied as a separation stage for Raman and mid-infrared spectroscopy.^13^

Recently, joint efforts of Jarrold and Clemmer groups at Indiana University have yielded a new approach to studying intact EVs (i.e., EVs with structurally undamaged membranes).^14,15^ In particular, the intact EVs were characterized using charge detection mass spectrometry (CDMS) with nanoelectrospray.^14^ The precisely measured charge and mass-to-charge ratio *m*/*z* of the EVs transported into the vacuum and characterized by the charge detector provided exact mass and charge distributions, with the last one reflecting structural and surface information. CDMS enabled distinguishing keratinocytic exosomes in diabetic and nondiabetic mice based on the charge distribution subpopulations retrieved by a Gaussian mixture model.^15^ The CDMS instrument used in these studies consisted of multipole nonresolving ion guides, acting simultaneously as a high-pass filter to transfer charged particles generated in nanoelectrospray from atmospheric pressure toward a multipass charge detector in vacuum.^14,16^

In other investigations, charged particles with *m*/*z* > 10^5^ were also analyzed using electrodynamic traps, including several Paul’s designs^17^ and linear quadrupole traps.^18−20^ The former were shown to be suitable for the analyses of high-mass microparticles, e.g., polystyrene particles and cell particles.^21−24^ It is noteworthy that MS using transmission linear quadrupoles is generally limited to *m*/*z* < 10^5^ with usually subunit resolution.^25^ The resolving power can reach thousands, albeit at the cost of ion transmission. For even higher *m*/*z*, the quadrupole driver electronics must feature extraordinary specifications, and a few special approaches to the quadrupole operation were introduced, i.e., the frequency-swept,^26^ frequency and amplitude scanned,^27^ and digitally driven^28−30^ modes. All rely on the frequency variation of the periodic waveform applied to pairs of quadrupole rods. The ‘frequency scanned’ mode utilizes a constant or semiconstant ratio of AC and DC components at a selected frequency to establish a stability zone for a certain *m*/*z*. In the digitally driven mode, the stability zone is defined by varied frequency and duty cycles of square waveforms without a DC component. In the case of linear ion traps, frequency of an axial or radial excitation periodic potential may be scanned to resonantly eject ions with different *m*/*z*.^31,32^

Here, we use the term ‘frequency-controlled’ because for the present application only the frequency of AC voltage is changed, and the resolution is defined by the ratio of constant AC and DC components. While quadrupoles are used in this work at fixed *m*/*z* settings, a frequency scan is necessary to obtain a mass spectrum.

The Selected Ice Nanoparticle Accelerator (SELINA) instrument was originally designed to produce mass-selected beams of highly charged water ice nanoparticles with 50–1000 nm diameter mimicking space dust found in the vicinity of several icy moons in our Solar system. Impact mass spectrometry of such particles accelerated electrostatically to hypervelocity is supposed to be the best terrestrial analogue experiment for past and future space missions with impact ionization MS instrumentation onboard. Moreover, it is the first step toward developing novel, high-resolution space probes based on impact ionization MS.^33^ The SELINA design is also suitable for the production and mass measurement of submicrometer solid particles from liquid samples nebulized by electrospray. Special care must be taken to fully desolve the particles. This procedure was successfully carried out for 300 and 600 nm polystyrene beads during the charge detector validation experiments.^34^

In this Technical Note, we showcase the operation of the SELINA apparatus when the resolving mode of the frequency-controlled quadrupole in the range of *m*/*z* 10^5^–10^6^ is used to identify different mass (size) of LUV fractions upon electrospraying a liquid sample of LUVs with a broad distribution. After quadrupole separation, positively charged LUVs were detected using a single-pass charge detector, which provided *m*/*z*, charge, and mass of an individual vesicle. Finally, in a proof-of-concept experiment, the technique was adopted to investigate a sample of the human blood serum, aiming to assess the underlying mass distribution fractions of bioparticles from the present heterogeneous sample. Considering the current advances in CDMS technology, i.e., electrostatic ion trap designs with a single elementary charge detection limit and superior resolution of *m*/*z*, charge, and mass, we deliberately use the ‘quadrupole mass sensor’ name for our setup to underline its application as a technique primarily used for vesicles separation from a sample, not for their thorough characterization, which could be otherwise possible by application of the state-of-the-art CDMS.

## Experimental Section

### SELINA Instrument

**Figure 1:**
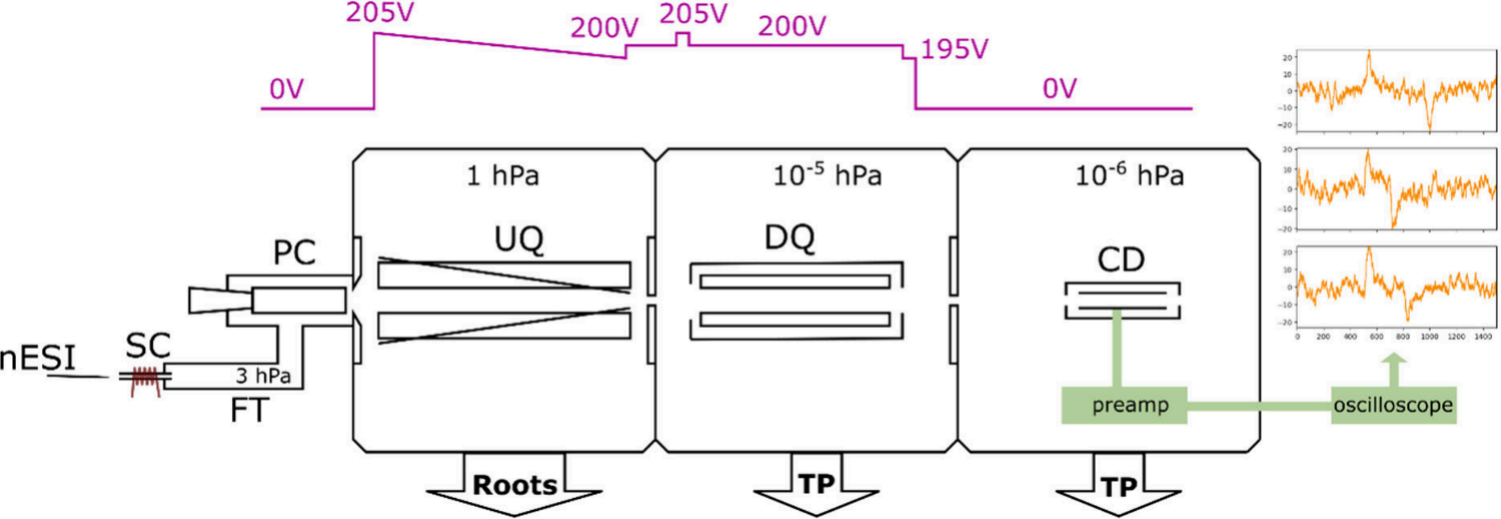
SELINA schematic representation with the main components denoted: nanoelectrospray emitter with the sample (nESI), 20 mm sampling capillary with resistive heating (SC), flow tube (FT), plenum chamber (PC), upstream quadrupole with slanted wires (UQ), downstream quadrupole (DQ), charge detector (CD), turbomolecular pumps (TP). The electric potential levels are shown at the top, and the exemplary signals from the CD, recorded by an oscilloscope, are on the right; the *x*-axis for signals is time in μs, and the *y*-axis is voltage in mV.

A detailed description of SELINA (shown schematically in Figure 1) has been published previously.^33^ Briefly, charged particles from a nanoelectrospray pass through a 20 mm long sampling capillary (SC) heated to approximately 200 °C for vesicle desolvation and continue moving through an aerodynamic system consisting of a flow tube with a plenum chamber (PC) by gradually losing their initial momentum in collisions with gas molecules. The particles are subsequently dragged by the jet of air into a first upstream quadrupole (UQ). This quadrupole is equipped with slanted wires between rods to impose an electric drift field (∼0.4 V cm^–1^) at a pressure of 1 hPa of lab air flowing through the PC. The charged particles are further thermalized in collisions with air molecules and drift in the UQ toward an orifice with a diameter of 1 mm into a differentially pumped chamber (5 × 10^–5^ hPa), which accommodates a linear quadrupole trap operated as a downstream quadrupole filter (DQ). The third differentially pumped chamber contains a single-pass charge detector (CD) to measure the particle’s charge and *m*/*z*. The charged particles leave the DQ through a 2 mm ID orifice and fly about 5 cm toward the 2 mm ID differential pumping orifice into the charge detector vacuum chamber and then an additional 5 cm toward the 2 mm ID entrance orifice into the charge detector. The potentials set were 205 V for the UQ, 195 V for the slanted wires, 205 V for the entrance DQ electrode, 200 V for the DQ and its enclosure, and 195 V for the exit DQ electrode.

### Charge Detector

The same CD as in ref (33) and ref (34) was utilized here, with the previously done charge calibration being 38 elementary charges (e) for 1 mV of the entrance peak amplitude of preamplifier response transient output for square pulse input. The procedure and corrections related to the shape of the transient signal and particle velocity are described in detail in ref (33). Briefly, the signal with amplitudes down to a few millivolts was pulsed through the calibration capacitor (0.8 pF), resulting in a minimum accessible injected charge of 20000 e. The linear dependence of the CD response on the injected charge is assumed valid down to the lower inaccessible values. This ‘low charge’ region was verified by polystyrene 300 nm beads measurement, where detected charges were in the range of 1000–5000 e. The CD electronics placed in a vacuum amplifies and shapes an image charge induced by a particle flying through a 50 mm long charge pick-up stainless steel tube. The resulting transients are recorded by an oscilloscope (PicoScope5000, Pico Technology, UK) at 16-bit resolution and sent to PC software for processing and storage. For vesicle measurement, the trigger was set to 20 mV, and the sampling rate and range were 50 kS/s and 1600 μs. Processed transients allow assessment of particle velocity (detector length divided by the time difference between entrance and exit peaks) and charge (average of amplitudes of entrance and exit peaks multiplied by 38 e/mV and velocity correction coefficient) and thus calculation of *m*/*z* (from known acceleration potential and velocity) and mass (as product of charge and *m*/*z*). An acceleration potential was defined by the potential of the DQ and was kept constant at 200 V.

The CD charge measurement error obtained from the blank signal is 328 e (see details in Figure S1), while the noise root-mean-square (RMS) is 131 e. Thus, for a particle with charge 600 e and *m*/*z* 2.5 × 10^5^, the relative standard deviation (RSD) is 66% for mass and 22% for diameter using charge measurement error 328 e. When noise is used, RSD values are 38% and 13%, respectively. The limit of charge detection of used CD is approximately 600 e, which is considerably higher than the usual value of 250 e for single-pass detectors.^35^ This is not an issue for the measurement of highly charged ice nanoparticles in SELINA, but just at the detection limit for vesicles.

### Quadrupoles

The mechanical assemblies of the ion-guiding UQ and the mass-resolving DQ were repurposed from commercial mass spectrometers (Thermo Scientific). The UQ has 250 mm long hyperbolic rods with a 6 mm field radius (distance from the axis to the rod), and the DQ is a part of the linear quadrupole trap assembly, which has 60 mm long hyperbolic rods and a 4.75 mm field radius. The DQ is enclosed and has entrance and exit electrodes. Its inner volume is filled at a flow rate of 1 sccm with lab air, which results in a pressure of 10^–3^–10^–2^ hPa in the enclosure. Both quadrupoles are powered by two dedicated voltage sources (JanasCard, Czechia) that can float at a potential of up to ± 1 kV. A direct digital synthesis chip is used to generate a harmonic signal with 0.03725 Hz resolution. The choice of harmonic potential simplifies the design of the sources and is advantageous in that *m*/*z* settings are calculated for the first stability region of the Mathieu diagram. The sources have slightly different operational parameters. The UQ source provides harmonic voltages in the 1–300 kHz range with a constant amplitude of 50 or 100 V, ensured via the automatic gain control loop. The DQ source operates in the 0.5–200 kHz with a constant nominal amplitude of 100 or 200 V corrected with the internal calibration table of peak-to-peak amplitudes depending on the frequency from 200.594 V at 500 Hz to 206.099 V at 200 kHz for the nominal amplitude 100 V, and from 401.862 V at 500 Hz to 400.437 V at 100 kHz for the nominal amplitude 200 V. The calibration table is sufficient for the quadrupole application presented here. However, high-precision measurement of the peak-to-peak voltage at each frequency would be necessary to obtain an exact *m*/*z* value of transmitted ions.^36^ Both sources provide variable DC components of 0 to ± 35 V with 16-bit resolution. The voltage parameters for certain *m*/*z* are calculated by a software (see below) accounting for the calibration table if necessary and sent to the voltage sources via USB. The *m*/*z* settings accessible for the present combination of quadrupole physical dimensions and voltage source characteristics allow to set *m*/*z* 1 × 10^3^–4 × 10^7^ for UQ and *m*/*z* 2 × 10^3^–4 × 10^8^ for DQ. The quadrupoles were used in two modes of operation: the nonresolving one (DC component for UQ and DQ was 0 V) and the resolving one (DC component for UQ was 0 V and ± 30.07 for DQ). For the sake of clarity, we will further mention only the *m*/*z* settings of DQ (*m*/*z*_DQ_), assuming that the UQ is always set to the same *m*/*z* with the DC component equal to 0 V. The DC component of the DQ for the resolving mode is calculated from the apex of the first stability region of the Mathieu diagram, being ∼0.168 of the AC amplitude and taken at the 90% percent tune. The voltage and frequency values for all *m*/*z* settings used are provided in Table S1 in the Supporting Information.

### LUVs Preparation

The suspension of LUVs was prepared as described below. The 100 μL aliquot of chloroform (Merck, Darmstadt, Germany) solution of POPC (1-palmitoyl-2-oleyl-*sn*-glycero-3-phosphocholine; Avanti Polar Lipids, Alabaster, AL) was transferred to a glass test tube. The solvent was evaporated under a stream of nitrogen, and the lipid film was kept under a vacuum overnight. The dry lipid film was hydrated with Mili-Q water (Millipore, USA). After 4 min of continuous vortexing, the suspension of multilamellar vesicles (MLVs) was extruded with a mini extruder (Avestin, Ottawa, Canada) through a polycarbonate membrane (Whatman; Little Chalfont, UK) with a nominal pore diameter of 200 nm. The lipid concentration was 1 mM, and the LUVs suspension was used at different dilutions.

The size distribution of LUVs suspension was measured using dynamic light scattering (DLS) setup Zetasizer Nano ZS (Malvern Instruments, Worcestershire, UK) consisting of a He–Ne laser (633 nm) and an avalanche photodiode detector (APD). To get the optimal scattering intensity, the sample was diluted to the 0.2 mM final lipid concentration, transferred to a plastic disposable cuvette (Brand, Wertheim, Germany), and measured after 2 min of equilibration time at 298 K. Scattered light was collected at the angle of 173° and intensity-weighted size distribution was obtained using Zetasizer Software 7.11.

### Nanoelectrospray Emitters Preparation and Vesicles Measurement

Nanoelectrospray emitters were pulled with the P-2000 laser-based micropipette puller (Sutter Instrument, CA) from the 10 cm length 1 mm OD, 0.78 mm ID borosilicate glass with filament (Sutter Instrument, BF100–78–10). The puller settings resulted mostly in a 4 mm taper with a closed tip. After the sample was loaded, the emitter was conditioned by gently touching the metal surface of the sampling capillary (SC) until the signal could be registered and no corona discharge was visible at a 1.5–2 mm distance from the SC. After the experiments, the emitters were inspected with a light microscope (Olympus FluoView 1000 MPE), as shown in Figure S2. The tip ID varied from 3 to 20 μm. The electrostatic potential was supplied to the platinum wire (0.05 mm diameter) within the emitter that was filled with the sample liquid. The emitters were first loaded with 300 nm latex beads in 100 mM ammonium acetate solution to find the suitable operating parameters of the nanoelectrospray (i.e., electric potential, distance toward SC, desolvation temperature, and the tip diameter) and to get rid of the solvent nanodroplets (larger than 50 nm in diameter). For 300 nm particles, the following parameters were found: the distance between the emitter and SC of 1.5–2 mm, the emitter potential of +1.2 kV and the SC temperature of 200 °C. Vesicle samples were then measured at identical conditions. After the latex beads sample was consumed, the sample with vesicles was loaded with a syringe into the emitter without changing its position and sprayed at the same potential. In this manner, polystyrene beads in a 100 mM ammonium acetate solution sample acted as a negative control, ensuring the absence of detectable solvent droplets at the defined nanoelectrospray conditions. The measurement procedure was repeated for three different emitters with freshly prepared vesicle samples each time (200 mM ammonium acetate aqueous solution and original vesicle sample 1:1 by volume). The transients recorded from the charge detector at different quadrupole settings were processed using Python with Numpy,^37^ Scipy,^38^ and Matplotlib^39^ libraries, and details are provided in the caption of Figure S4. Postprocessing was necessary to exclude invalid transients (e.g., when signals of two or more particles overlapped). While in the nonresolving mode with a high count rate, 49% of the transients had to be discarded, only 14% of transients were found invalid in the resolving mode having substantially lower count rates (see Table S2 for details). Processed results for all three emitter-sample couples are also provided in the SI (Figures S6–S9). Since the resulting *m*/*z*, charge, mass, and diameter distributions belonging to the different emitters did not differ substantially, we focus on the data acquired with the second emitter sample in the next section.

### Blood Serum Measurement

The 6 mL of blood was sampled from the veins of the volunteer into a blood-collection tube containing sodium citrate (final concentration of 3.8% w/V). The sample was left to separate erythrocytes (red blood cells) and platelets from serum by standing still at room temperature for 3 h. The upper yellow-brown supernatant was transferred into a new 15 mL tube with a conical bottom and centrifuged for 10 min at 1500*g* at room temperature to remove debris and white blood cells. The supernatant (blood serum) was transferred into 3 new 1.5 mL Eppendorf tubes and stored in a fridge at +8 °C for 5 days in a vertical position.

The 20 μL of serum was taken from the upper half of the vial and mixed with 800 μL of 100 mM ammonium acetate solution. The emitter distance from SC was about 2 mm and nanoelectrospray potential +1.3 kV. The size distribution of the blood serum sample was measured using the same DLS setup and experimental conditions as those used for the LUVs suspension. Before being transferred to a plastic disposable cuvette, the blood serum was diluted with water in a 1:1 (v/v) ratio.

## Results and Discussion

### LUVs Sample

**Figure 2:**
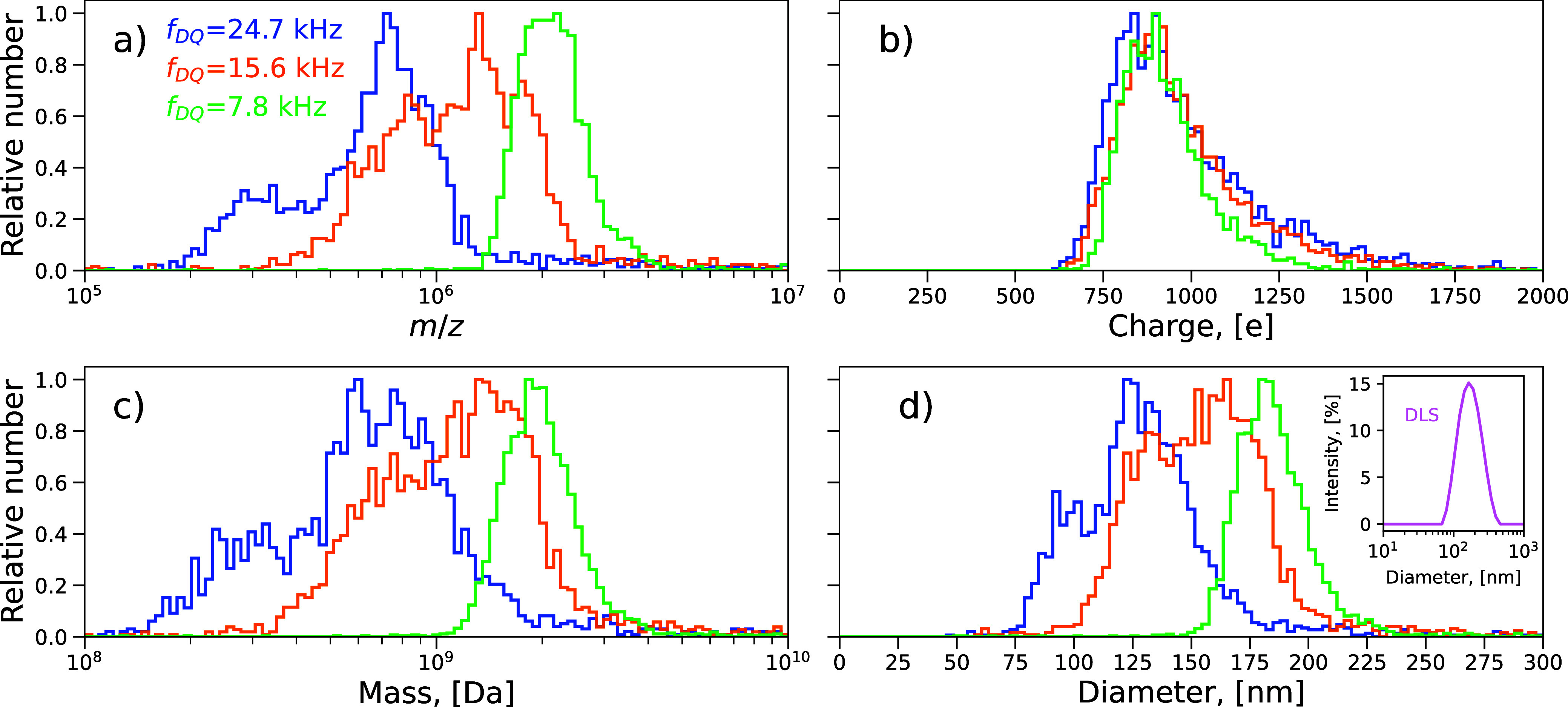
Histogram distributions of *m*/*z* (a), charge (b), mass (c), and diameter (d) of vesicles as measured by the charge detector at three different frequencies (color coded) of DQ in the nonresolving mode. The DLS measurement is plotted in panel (d) inset for comparison. The numbers of CD detection events used to plot histogram distributions are 2454, 2585, and 3141 for *f*_DQ_ values of 24.7, 15.6, and 7.8 kHz, respectively; bin number in each histogram is 100 with equal widths (b, d) and varying widths spaced evenly on a log scale (a, c).

After loading the emitter with the vesicle sample, the DQ in nonresolving mode was used to transfer all charged vesicles from nanoelectrospray toward the charge detector. To transmit the full range of LUVs with initially unknown *m*/*z* distribution, the DQ frequencies were set to 24.7, 15.6, and 7.8 kHz. These values correspond to *m*/*z*_DQ_ settings 2 × 10^5^, 5 × 10^5^, and 2 × 10^6^ with 0 DC component, respectively, and comply with the lower *m*/*z* limit in the stability diagram. Each frequency setting was repeated in cycles with 30 s dwell time for 40 min to collect a sufficient number of detection events while keeping enough amount of the sample in the emitter for the subsequent measurements in the resolving mode. Upon passage through the CD, the charged vesicle generates a transient signal. Once digitized, the signal allows us to obtain the charge and the velocity; the latter provides *m*/*z* for a known accelerating potential (i.e., DQ potential of 200 V). The mass was calculated as a product of the mass-to-charge ratio and the charge; the diameter of the vesicle was derived from the mass, assuming the spherical shape of LUVs and a density of water (997 kg/m^3^). All experimental and derived values of *m*/*z*, charge, mass, and diameter at each nonresolving DQ setting are plotted in Figure 2 as histogram distributions. Figure 2a shows that the vesicle *m*/*z* distributions roughly span the range from 2 × 10^5^ to 3 × 10^6^. Because of the close similarity of the three charge distributions (Figure 2b), the shape of the corresponding mass distributions resembles that of *m*/*z* (Figure 2c). In comparison with the DLS measurement (inset in Figure 2d), the diameter distributions are slightly shifted to lower values, but most abundant diameters are in the same range between 100 and 200 nm.

**Figure 3:**
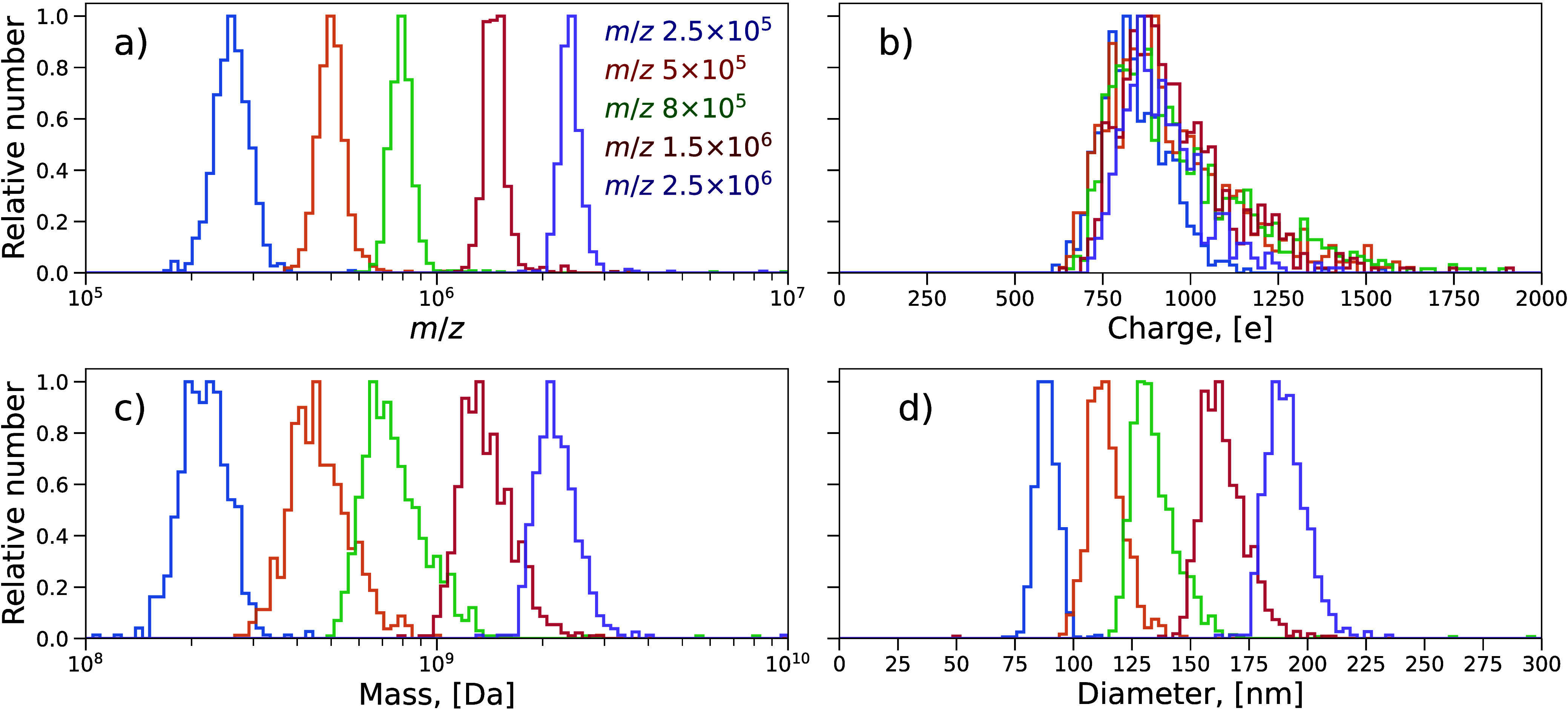
Histogram distributions of *m*/*z* (a), charge (b), mass (c), and diameter (d) of vesicles as determined by the charge detector with the DQ quadrupole in a resolving mode at five different *m*/*z* settings (color-coded). Numbers of CD detection events used to plot histogram distributions are 646, 705, 897, 748, and 503 for *m*/*z*_DQ_ 2.5 × 10^5^, 5 × 10^5^, 8 × 10^5^, 1.5 × 10^6^, and 2.5 × 10^6^ respectively; bin number in each histogram is 100 with equal widths (b, d) and varying widths spaced evenly on a log scale (a, c).

To acquire separate narrower mass distributions of LUVs, the DQ was used in resolving mode, and five specific *m*/*z*_DQ_ were selected from the range obtained in the nonresolving mode. The resulting *m*/*z*, charge, mass, and diameter distributions are shown in Figure 3. Again, due to the similarity of charge distributions (Figure 3b), the mass distributions (Figure 3c) are determined primarily by that of *m*/*z* (Figure 3a). The *m*/*z* resolving power calculated as a ratio of the *m*/*z* peak position and its Full Width at Half Maximum (fwhm) is about 5 and did not change substantially at higher DQ resolution settings (see Figure.S10). This means, in turn, that the peak fwhm is defined not by the quadrupole but by the error of the *m*/*z* measurement with the charge detector. The relative standard deviation (RSD) can be estimated from Gaussian fits to *m*/*z* distributions, and it ranges from 12% for *m*/*z* 2.5 × 10^5^ to 7% for *m*/*z* 2.5 × 10^6^.

The charge distributions at different *m*/*z*_DQ_ settings are generally similar, with their modal values in the range of 750–900 e, and various tailings toward higher charges. The detection limit of the present CD is about 600 e, as mentioned in the Experimental section, and careful signal processing was required, especially when several charged particles were recorded simultaneously in one transient (see Figure S4). Additionally, heatmaps of the charge versus mass dependence are shown in Figure S12 for both nonresolving and resolving modes of operation. All charges measured are smaller than the Rayleigh charge limit for water, and their number does not show any substantial increase with the diameter (mass). On the other hand, distinct charge distributions may indeed be observed with CDMS. This was the case in the studies of lipoproteins and exosomes, where different particle structures, geometries, and molecular cargos located on the surface resulted in several distinct charge subpopulations.^15,40^ The reduction of a charge of electrosprayed spherical-shaped bacterium *Staphylococcus aureus* (500–1000 nm) with increasing sampling capillary temperature was explored by Shao-Yu Liang et al. using a charge-sensing particle detector.^41^ They discovered that at 200 °C, the bacterium was fully desolvated from methanol and bears a mean charge of 1046 e, which is compatible with values obtained here.

**Figure 4:**
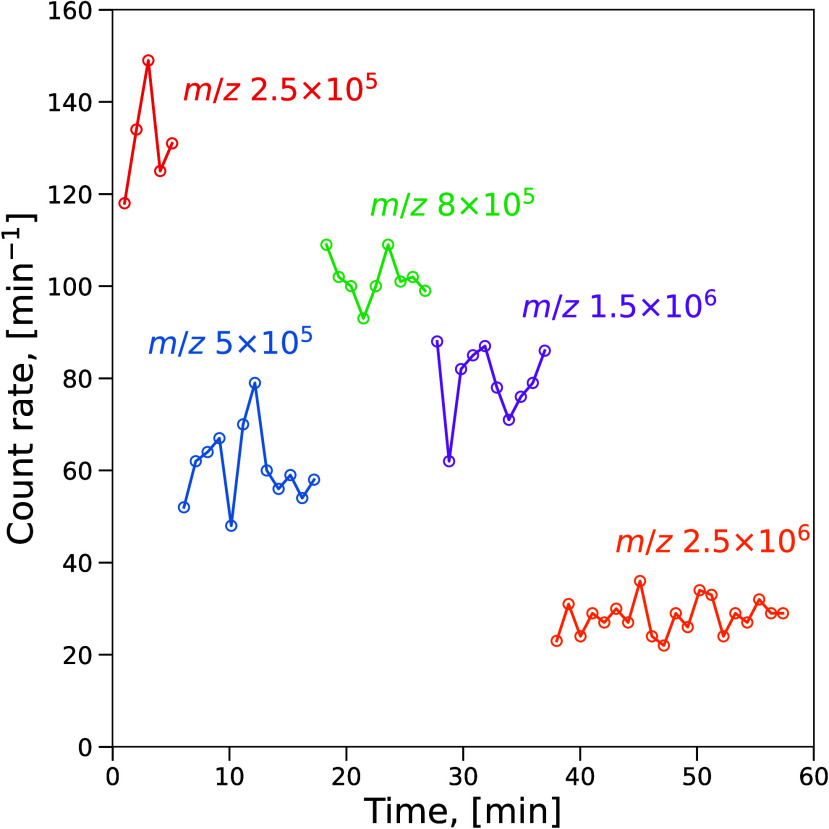
Measurement time series of the vesicle count rate as detected by the charge detector at various *m*/*z* settings of DQ in resolving mode.

Figure 4 shows the time series of the vesicle detection count rate in the resolving mode for five *m*/*z*_DQ_ settings. Though a gas-filled DQ focuses charged vesicles on the instrument axis, some divergence is expected downstream so that the number of detection events is lower compared to the number of vesicles leaving the DQ. Assuming an average flow rate in the nanoelectrospray being 50 nL min^–1^, we can roughly estimate the total number of vesicles emitted in front of the SC as 10^8^ per minute (see SI for the calculation details). It is, however, not clear how many of them were sampled into SELINA.

### Blood Serum Sample

The measurement of the blood serum was done in the same manner as that for the LUVs samples. First, the DQ was used in the nonresolving mode to get broad *m*/*z* distributions of charged particles at *f*_DQ_ values of 24.7 kHz, 15.6 kHz, and 7.8 kHz. The *m*/*z* values of detected particles during these initial measurements with different emitters were roughly in the 10^5^–10^7^ range (see Figure S14). The detection of particles with *m*/*z* > 10^6^ at *f*_DQ_ = 7.8 kHz was unstable and occurred in short bursts, probably affected by some processes in the nanoelectrospray ion source. Unlike LUVs, the DLS analysis of the serum sample shows a much wider, multimodal size distribution with diameters from 4 nm to 6 μm featuring three peaks around 10, 40, and 500 nm (see Figure S13).

**Figure 5:**
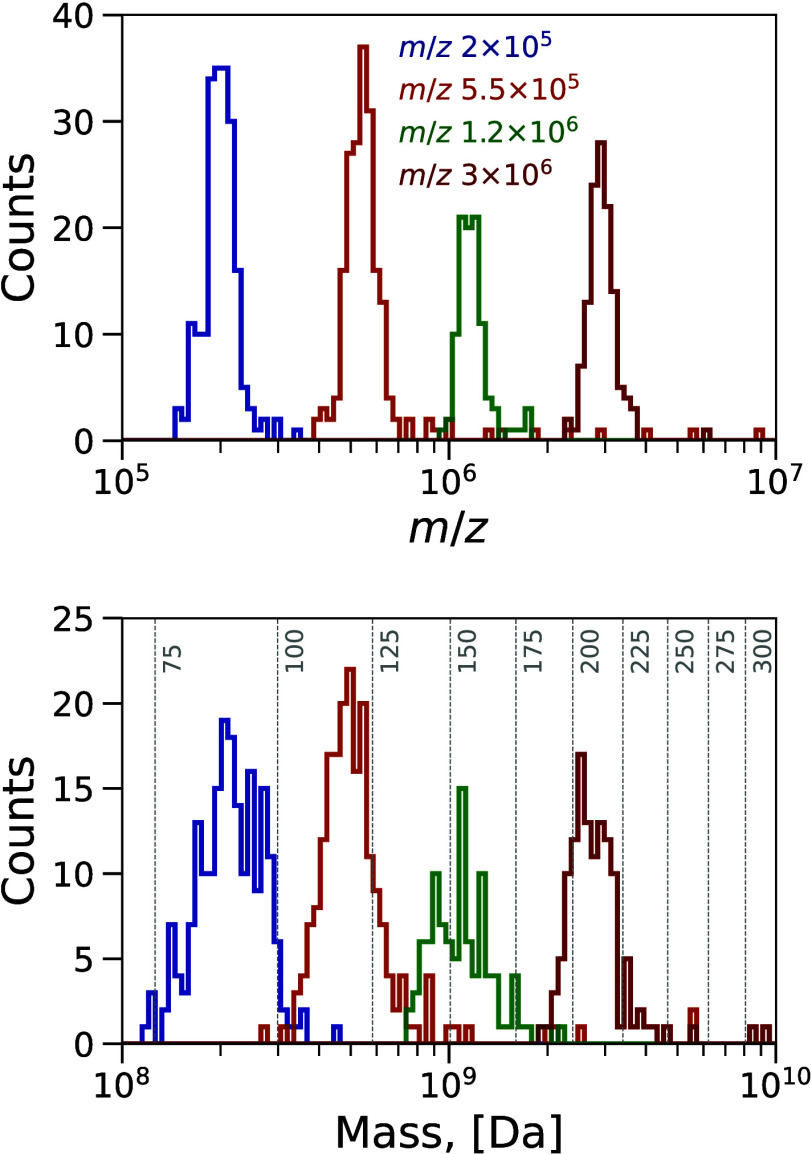
Histogram distributions of *m*/*z* (top) and mass (bottom) for serum sample particles as detected by CD at *m*/*z*_DQ_ 2 × 10^5^, 5.5 × 10^5^, 1.2 × 10^6^, 3 × 10^6^. In the bottom plot, the gray vertical lines denote mass values equivalent to spherical particles with a density of 997 kg m^–3^ for different diameters in nanometers. The bin number in each histogram is 100, with varying widths spaced evenly on a log scale.

In the resolving mode, DQ was set to six different values of *m*/*z*_DQ_ (i.e., 2 × 10^5^, 3 × 10^5^, 5.5 × 10^5^, 7 × 10^5^, 1.2 × 10^6^, 3 × 10^6^) based on *m*/*z* distributions obtained in the nonresolving mode. While Figure 5 shows histograms for *m*/*z* and mass obtained with the CD at four *m*/*z*_DQ_ values (i.e., 2 × 10^5^, 5.5 × 10^5^, 1.2 × 10^6^, 3 × 10^6^), all of them are depicted in Figure S15. The diameter distributions are not evaluated here, as the nature of the detected particles and thus their shape and densities are not known. Instead masses of spherical water particles with various diameters are depicted in Figure 5 for the sake of comparison. The count rates in the resolving mode were lower than in the LUVs experiment and covered the range from 6 min^–1^ at *m*/*z*_DQ_ 3 × 10^6^ to 30 min^–1^ at *m*/*z*_DQ_ 2 × 10^5^. We also observed that after about 30 min, a visible film was formed near the orifice of the sampling capillary and partial clogging of the latter started after an even longer period of operation.

## Conclusions and Outlook

We have demonstrated that LUVs with a 100–200 nm diameter can be charged in nanoelectrospray from aqueous solutions, transferred into the vacuum, and thermalized to a defined electric potential in the SELINA instrument. The combination of two wide *m*/*z*-range frequency-controlled quadrupoles, where the first one was used in nonresolving mode while the second one in resolving mode, facilitates the mass filtering of vesicles based on their *m/z.* As a result, we have succeeded in separating a broad initial distribution (fwhm of 200 nm, see Figure 2d) into distinct LUVs fractions having fwhm of 20–30 nm. The charged LUVs were mass characterized by a single-pass charge detector operated in the current study near its detection limit of 600 e. In future investigations, we will apply an improved design of the charge detector based on electrostatic trapping with a detection limit down to tens of elementary charges.^42^ It will enhance the precision of the mass measurements and allow us, for instance, to investigate smaller vesicles.

It is well established that real biological samples containing EVs feature a high degree of particle (vesicle) heterogeneity. Therefore, both *m*/*z* and the mass correspondence may not be unique due to the broad charge and density distributions of the bioparticles under study. Nevertheless, quadrupole preseparation performed in this work can be used to select a relatively narrow mass fraction for a detailed mass spectrometric characterization of the content carried by the selected vesicles. If needed, a deflection stage synchronized with the charge detector can be added to single out particles with a desired mass or charge as it was applied in dust particle accelerators.^43^

The present pilot investigation with blood serum demonstrated that quadrupole-based mass separation of particles from a highly heterogeneous sample is indeed feasible. We found out that separation efficiency was not compromised due to the broader charge distribution of the blood serum sample with respect to LUVs. Currently, the main limitations are the stability of the nanoelectrospray source and a low count rate of the separated particles in resolving mode. In particular, for larger particles the count rate achieved was only 6 min^–1^. The modifications of the ion source, atmospheric pressure interface, and use of additional ion optics could help to increase the count rate at least several times. Moreover, lower quadrupole resolution settings could further increase the transfer rate of vesicles, although the resulting size distribution will be broader.

Comparing our findings to those obtained by other separation techniques so far, the obvious advantage of the presented approach is its selectivity since the mass and charge are measured for each individual particle. However, we consider the short (millisecond) time scale needed for separation and mass characterization of an individual vesicle to be the main benefit of our approach. It enables a direct and contamination-free selection of vesicles that can be subjected to additional analyses. Particularly, in an electrodynamic trap placed downstream, selected vesicles could be accumulated and irradiated by short mid-IR laser pulses inducing the desorption of (charged) proteins and peptides from the vesicle membrane,^44^ which can then be analyzed using e.g., a TOF mass spectrometer. A dedicated power supply allowing simultaneous storage of both the vesicles and desorbed species would be necessary for such a trap.^19^ For example, to trap ions with *m*/*z* from 200 to 2 × 10^6^ in a linear quadrupole trap with a 0.475 cm effective radius, a harmonic voltage with frequency of 1 MHz and an amplitude of 400 V would be required.^45^ To this end, the count rate of 26 min^–1^ achieved for the serum sample at *m*/*z*_DQ_ 5.5 × 10^5^ makes us confident that such trapping experiment coupled with desorption MS analysis may be worth trying. Our future development of the current concept will therefore aim at the separation of individual EVs from real biomedical samples, with the main objective of obtaining EVs’ signatures (e.g., mass and charge distributions) together with the mass spectra of cargo proteins.

## Data Availability

The data that support the findings of this study are openly available in the National Data Repository at 10.48700/datst.hqpbx-8ws91.

## Supporting Information Available

Histogram plots for charge measurement error; charge detector measurement error; table with quadrupole settings; microphotography of nanoelectrospray emitters; DLS analysis of 200 nm vesicle sample; examples of the charge detector signal postprocessing; table with samples comparison and summary of the validation; histogram distributions for 300 nm polystyrene beads; histogram distributions for vesicles in nonresolving mode; density histogram distributions of vesicles diameter in nonresolving mode; histogram distributions of vesicles in resolving mode; density histogram distributions of vesicles diameter in resolving mode; comparison of the different *m*/*z* histogram distributions; measurement time series of the vesicle count rate in nonresolving mode; lipid vesicles number calculation; heatmaps for the charge vs mass for vesicles; DLS analysis of the blood serum sample; histogram distributions for serum samples in nonresolving mode; histogram distributions for serum samples in resolving mode (PDF)

## References

[ref1] Welsh J. A.; Goberdhan D. C.; O’Driscoll L.; Buzas E.; Blenkiron C.; Bussolati B.; Cai H.; Di Vizio D.; Driedonks T. A. P.; Erdbrügger U.; Falco-Perez J. M.; Fu Q. L.; Hill A. F.; Lenassi M.; Lim S. K.; Mahoney M. G.; Mohanty S.; Möller A.; Nieuwland R.; Witwer K. W. Minimal information for studies of extracellular vesicles (MISEV2023): From basic to advanced approaches. J. Extracell Vesicles 2024, 13 (2), e12404 10.1002/jev2.12404. 38326288 PMC10850029

[ref2] Meldolesi J. Exosomes and Ectosomes in Intercellular Communication. Curr. Biol. 2018, 28 (8), R435–R444. 10.1016/j.cub.2018.01.059. 29689228

[ref3] Ciferri M. C.; Quarto R.; Tasso R. Extracellular Vesicles as Biomarkers and Therapeutic Tools: From Pre-Clinical to Clinical Applications. Biology-Basel 2021, 10 (5), 359 10.3390/biology10050359. 33922446 PMC8145169

[ref4] Mathew B.; Mansuri M. S.; Williams K. R.; Nairn A. C. Exosomes as Emerging Biomarker Tools in Neurodegenerative and Neuropsychiatric Disorders-A Proteomics Perspective. Brain Sci. 2021, 11 (2), 258 10.3390/brainsci11020258. 33669482 PMC7922222

[ref5] Pocsfalvi G.; Stanly C.; Vilasi A.; Fiume I.; Capasso G.; Turiák L.; Buzas E. I.; Vékey K. Mass spectrometry of extracellular vesicles. Mass Spectrom Rev. 2016, 35 (1), 3–21. 10.1002/mas.21457. 25705034

[ref6] Askeland A.; Borup A.; Ostergaard O.; Olsen J. V.; Lund S. M.; Christiansen G.; Kristensen S. R.; Heegaard N. H. H.; Pedersen S. Mass-Spectrometry Based Proteome Comparison of Extracellular Vesicle Isolation Methods: Comparison of ME-kit, Size-Exclusion Chromatography, and High-Speed Centrifugation. Biomedicines 2020, 8 (8), 246 10.3390/biomedicines8080246. 32722497 PMC7459681

[ref7] van der Pol E.; Coumans F. A. W.; Grootemaat A. E.; Gardiner C.; Sargent I. L.; Harrison P.; Sturk A.; van Leeuwen T. G.; Nieuwland R. Particle size distribution of exosomes and microvesicles determined by transmission electron microscopy, flow cytometry, nanoparticle tracking analysis, and resistive pulse sensing. J. Thromb Haemost 2014, 12 (7), 1182–1192. 10.1111/jth.12602. 24818656

[ref8] van Niel G.; D’Angelo G.; Raposo G. Shedding light on the cell biology of extracellular vesicles. Nat. Rev. Mol. Cell Bio 2018, 19 (4), 213–228. 10.1038/nrm.2017.125. 29339798

[ref9] Piontek M. C.; Lira R. B.; Roos W. H. Active probing of the mechanical properties of biological and synthetic vesicles. Bba-Gen Subjects 2021, 1865 (4), 129486 10.1016/j.bbagen.2019.129486. 31734458

[ref10] Nikoloff J. M.; Saucedo-Espinosa M. A.; Kling A.; Dittrich P. S. Identifying extracellular vesicle populations from single cells. P Natl. Acad. Sci. USA 2021, 118 (38), e2106630118 10.1073/pnas.2106630118. PMC8463870 34518226

[ref11] van der Koog L.; Gandek T. B.; Nagelkerke A. Liposomes and Extracellular Vesicles as Drug Delivery Systems: A Comparison of Composition, Pharmacokinetics, and Functionalization. Adv. Healthc Mater. 2022, 11 (5), 2100639 10.1002/adhm.202100639. 34165909 PMC11468589

[ref12] Cebecauer M.; Amaro M.; Jurkiewicz P.; Sarmento M. J.; Sachl R.; Cwiklik L.; Hof M. Membrane Lipid Nanodomains. Chem. Rev. 2018, 118 (23), 11259–11297. 10.1021/acs.chemrev.8b00322. 30362705

[ref13] Weiss V. U.; Wieland K.; Schwaighofer A.; Lendl B.; Allmaier G. Native Nano-electrospray Differential Mobility Analyzer (nES GEMMA) Enables Size Selection of Liposomal Nanocarriers Combined with Subsequent Direct Spectroscopic Analysis. Anal. Chem. 2019, 91 (6), 3860–3868. 10.1021/acs.analchem.8b04252. 30735037 PMC6427476

[ref14] Brown B. A.; Zeng X. Y.; Todd A. R.; Barnes L. F.; Winstone J. M. A.; Trinidad J. C.; Novotny M. V.; Jarrold M. F.; Clemmer D. E. Charge Detection Mass Spectrometry Measurements of Exosomes and other Extracellular Particles Enriched from Bovine Milk. Anal. Chem. 2020, 92 (4), 3285–3292. 10.1021/acs.analchem.9b05173. 31989813 PMC7236431

[ref15] Brown B. A.; Guda P. R.; Zeng X. Y.; Anthony A.; Couse A.; Barnes L. F.; Sharon E. M.; Trinidad J. C.; Sen C. K.; Jarrold M. F.; Ghatak S.; Clemmer D. E. Analysis of Keratinocytic Exosomes from Diabetic and Nondiabetic Mice by Charge Detection Mass Spectrometry. Anal. Chem. 2022, 94 (25), 8909–8918. 10.1021/acs.analchem.2c00453. 35699514 PMC9450994

[ref16] Todd A. R.; Jarrold M. F. Dramatic Improvement in Sensitivity with Pulsed Mode Charge Detection Mass Spectrometry. Anal. Chem. 2019, 91 (21), 14002–14008. 10.1021/acs.analchem.9b03586. 31589418 PMC6834878

[ref17] Xiong C. Q.; Liu H. H.; Li Y. Z.; Meng L. W.; Wang J. Y.; Nie Z. X. High Speed Mass Measurement of a Single Metal-Organic Framework Nanocrystal in a Paul Trap. Anal. Chem. 2022, 94 (6), 2686–2692. 10.1021/acs.analchem.1c03845. 35112854

[ref18] Lee J.; Marino M. A.; Koizumi H.; Reilly P. T. A. Simulation of duty cycle-based trapping and ejection of massive ions using linear digital quadrupoles: The enabling technology for high resolution time-of-flight mass spectrometry in the ultra high mass range. Int. J. Mass Spectrom. 2011, 304 (1), 36–40. 10.1016/j.ijms.2011.03.011. 21731427 PMC3126150

[ref19] Bhanot J. S.; Fabijanczuk K. C.; Abdillahi A. M.; Chao H. C.; Pizzala N. J.; Londry F. A.; Dziekonski E. T.; Hager J. W.; McLuckey S. A. Adaptation and operation of a quadrupole/time-of-flight tandem mass spectrometer for high mass ion/ion reaction studies. Int. J. Mass Spectrom. 2022, 478, 116874 10.1016/j.ijms.2022.116874. 37032994 PMC10081487

[ref20] Patil A. A.; Liu Z.-X.; Chiu Y.-P.; Lai T. K. L.; Chou S.-W.; Cheng C.-Y.; Su W.-M.; Liao H.-T.; Agcaoili J. B. A.; Peng W.-P. Development of a linear ion trap mass spectrometer capable of analyzing megadalton MALDI ions. Talanta 2023, 259, 124555 10.1016/j.talanta.2023.124555. 37088041

[ref21] Schlunegger U. P.; Stoeckli M.; Caprioli R. M. Frequency scan for the analysis of high mass ions generated by matrix-assisted laser desorption/ionization in a Paul trap. Rapid Commun. Mass Sp 1999, 13 (18), 1792–1796. 10.1002/(SICI)1097-0231(19990930)13:18<1792::AID-RCM715>3.0.CO;2-S. 10482890

[ref22] Peng W. P.; Lin H. C.; Chu M. L.; Chang H. C.; Lin N. H.; Yu A. L.; Chen C. H. Charge monitoring cell mass spectrometry. Anal. Chem. 2008, 80 (7), 2524–2530. 10.1021/ac7024392. 18321134

[ref23] Zhu Z. Q.; Xiong C. Q.; Xu G. P.; Liu H.; Zhou X. Y.; Chen R.; Peng W. P.; Nie Z. X. Characterization of bioparticles using a miniature cylindrical ion trap mass spectrometer operated at rough vacuum. Analyst 2011, 136 (7), 1305–1309. 10.1039/c0an00911c. 21305099

[ref24] Nie Z.; Cui F.; Tzeng Y. K.; Chang H. C.; Chu M.; Lin H. C.; Chen C. H.; Lin H. H.; Yu A. L. High-speed mass analysis of whole erythrocytes by charge-detection quadrupole ion trap mass Spectrometry. Anal. Chem. 2007, 79 (19), 7401–7407. 10.1021/ac071207e. 17784735

[ref25] Douglas D. J. Linear Quadrupoles in Mass Spectrometry. Mass Spectrom Rev. 2009, 28 (6), 937–960. 10.1002/mas.20249. 19492304

[ref26] Marmet P.; Proulx M. A Frequency-Swept Quadrupole Mass Filter. Int. J. Mass Spectrom. 1982, 42 (1–2), 3–10. 10.1016/0020-7381(82)80047-8.

[ref27] Shinholt D. L.; Anthony S. N.; Alexander A. W.; Draper B. E.; Jarrold M. F. A frequency and amplitude scanned quadrupole mass filter for the analysis of high m/z ions. Rev. Sci. Instrum. 2014, 85 (11), 113109 10.1063/1.4900627. 25430100

[ref28] Opacic B.; Hoffman N. M.; Clowers B. H.; Reilly P. T. A. Impact of injection potential on measured ion response for digitally driven mass filters. Int. J. Mass Spectrom. 2018, 434, 1–6. 10.1016/j.ijms.2018.08.009.

[ref29] Simke F.; Fischer P.; Marx G.; Schweikhard L. Simulations of a digital ion filter and a digital ion trap for heavy biomolecules. Int. J. Mass Spectrom. 2022, 473, 116779 10.1016/j.ijms.2021.116779.

[ref30] Hu R.; Gundlach-Graham A. Simulation study of digital waveform-driven quadrupole mass filter operated in higher stability regions for high-resolution inductively coupled plasma mass spectrometry. Rapid Commun. Mass Sp 2024, 38 (12), e9753 10.1002/rcm.9753. 38616299

[ref31] Remes P. M.; Syka J. E. P.; Kovtoun V. V.; Schwartz J. C. Insight into the Resonance Ejection Process during Mass Analysis through Simulations for Improved Linear Quadrupole Ion Trap Mass Spectrometer Performance (Reprinted). Int. J. Mass Spectrom. 2015, 377, 368–384. 10.1016/j.ijms.2014.08.014.

[ref32] Londry F. A.; Hager J. W. Mass selective axial ion ejection from a linear quadrupole ion trap. J. Am. Soc. Mass Spectrom. 2003, 14 (10), 1130–1147. 10.1016/S1044-0305(03)00446-X. 14530094

[ref33] Spesyvyi A.; Zabka J.; Polásek M.; Charvat A.; Schmidt J.; Postberg F.; Abel B. Charged Ice Particle Beams with Selected Narrow Mass and Kinetic Energy Distributions. J. Am. Soc. Mass Spectrom. 2023, 34 (5), 878–892. 10.1021/jasms.2c00357. 37018538

[ref34] Spesyvyi A.; Zabka J.; Polasek M.; Maleckova M.; Khawaja N.; Schmidt J.; Kempf S.; Postberg F.; Charvat A.; Abel B. Selected ice nanoparticle accelerator hypervelocity impact mass spectrometer (SELINA-HIMS): features and impacts of charged particles. Philos. T R Soc. A 2024, 382 (2273), 20230208 10.1098/rsta.2023.0208. 38736336

[ref35] Keifer D. Z.; Pierson E. E.; Jarrold M. F. Charge detection mass spectrometry: weighing heavier things. Analyst 2017, 142 (10), 1654–1671. 10.1039/C7AN00277G. 28443838

[ref36] Peng W. P.; Lee Y. T.; Ting J. W.; Chang H. C. Averaging peak-to-peak voltage detector for absolute mass determination of single particles with quadrupole ion traps. Rev. Sci. Instrum. 2005, 76 (2), 023108 10.1063/1.1841791.

[ref37] Harris C. R.; Millman K. J.; van der Walt S. J.; Gommers R.; Virtanen P.; Cournapeau D.; Wieser E.; Taylor J.; Berg S.; Smith N. J.; Kern R.; Picus M.; Hoyer S.; van Kerkwijk M. H.; Brett M.; Haldane A.; del Río J. F.; Wiebe M.; Peterson P.; Gérard-Marchant P.; Sheppard K.; Reddy T.; Weckesser W.; Abbasi H.; Gohlke C.; Oliphant T. E. Array programming with NumPy. Nature 2020, 585 (7825), 357–362. 10.1038/s41586-020-2649-2. 32939066 PMC7759461

[ref38] Virtanen P.; Gommers R.; Oliphant T. E.; Haberland M.; Reddy T.; Cournapeau D.; Burovski E.; Peterson P.; Weckesser W.; Bright J.; van der Walt S. J.; Brett M.; Wilson J.; Millman K. J.; Mayorov N.; Nelson A. R. J.; Jones E.; Kern R.; Larson E.; Carey C. J.; Polat I.; Feng Y.; Moore E. W.; VanderPlas J.; Laxalde D.; Perktold J.; Cimrman R.; Henriksen I.; Quintero E. A.; Harris C. R.; Archibald A. M.; Ribeiro A. N. H.; Pedregosa F.; van Mulbregt P.; et al. SciPy 1.0: fundamental algorithms for scientific computing in Python. Nat. Methods 2020, 17 (3), 261–272. 10.1038/s41592-019-0686-2. 32015543 PMC7056644

[ref39] Hunter J. D. Matplotlib: A 2D graphics environment. Comput. Sci. Eng. 2007, 9 (3), 90–95. 10.1109/MCSE.2007.55.

[ref40] Lutomski C. A.; Gordon S. M.; Remaley A. T.; Jarrold M. F. Resolution of Lipoprotein Subclasses by Charge Detection Mass Spectrometry. Anal. Chem. 2018, 90 (11), 6353–6356. 10.1021/acs.analchem.8b01127. 29756771

[ref41] Liang S. Y.; Estayan M. I. C.; Hsieh L. W.; Pan M. C.; Li K. X.; Chang H. C.; Peng W. P. Real-Time Monitoring of the Evaporation and Fission of Electrospray-Ionized Polystyrene Beads and Bacterial Pellets at Elevated Temperatures. Anal. Chem. 2024, 96 (18), 7179–7186. 10.1021/acs.analchem.4c00763. 38661266

[ref42] Hogan J. A.; Jarrold M. F. Optimized Electrostatic Linear Ion Trap for Charge Detection Mass Spectrometry. J. Am. Soc. Mass Spectrom. 2018, 29 (10), 2086–2095. 10.1007/s13361-018-2007-x. 29987663

[ref43] Shu A.; Collette A.; Drake K.; Grün E.; Horányi M.; Kempf S.; Mocker A.; Munsat T.; Northway P.; Srama R.; Sternovsky Z.; Thomas E. 3 MV hypervelocity dust accelerator at the Colorado Center for Lunar Dust and Atmospheric Studies. Rev. Sci. Instrum. 2012, 83 (7), 075108 10.1063/1.4732820. 22852725

[ref44] Hellwig N.; Martin J.; Morgner N. LILBID-MS: using lasers to shed light on biomolecular architectures. Biochem Soc. T 2022, 50 (3), 1057–1067. 10.1042/BST20190881. PMC9317959 35695670

[ref45] Gerlich D. Inhomogeneous Rf-Fields - a Versatile Tool for the Study of Processes with Slow Ions. Adv. Chem. Phys. 1992, 82, 1–176. 10.1002/9780470141397.ch1.

