# Supplementary material for: Separation and Detection of Charged Unilamellar Vesicles in Vacuum by a Frequency-Controlled Quadrupole Mass Sensor
Source: Anal Chem. 2025 Apr 22;97(17):9131–8. doi: 10.1021/acs.analchem.4c05730 (PMC12060092; doi:10.1021/acs.analchem.4c05730)
Supplement: Supplementary file 1 — ac4c05730_si_001.pdf [file ac4c05730_si_001.pdf]

Supporting Information

Separation and Detection of Charged Unilamellar Vesicles in Vacuum by a Frequency-Controlled Quadrupole Mass Sensor

Anatolii Spesyvyi[1]*, Marek Cebecauer[1], Ján Žabka[1], Agnieszka Olżyńska[1], Michaela Malečková[1], Zuzana Johanovská[1,2], Miroslav Polášek[1], Ales Charvat[3,4], Bernd Abel[1,3,4]

[1] J. Heyrovský Institute of Physical Chemistry of the Czech Academy of Sciences, Prague 18223, Czechia

[2] Faculty of Mathematics and Physics, Charles University, Prague 12116, Czechia

[3] Institute of Chemical Technology, Leipzig University, Leipzig 04103, Germany

[4] Leibniz Institute of Surface Engineering, Leipzig 04318, Germany

*Email: anatolii.spesyvyi@jh-inst.cas.cz

Table of Contents

Figure S1. Histogram plots for charge measurement error S3

Charge detector measurement error S3

Table S1 Quadrupoles settings S4

Figure S2. Microphotography of nanoelectrospray emitters S5

Figure S3. DLS analysis of 200 nm vesicle sample S5

Figure S4. Examples of the charge detector signal postprocessing S6

Table S2 Samples comparison and summary of the validation S7

Figure S5. Histogram distributions for 300 nm polystyrene beads S8

Figure S6. Histogram distributions for vesicles in nonresolving mode S9

Figure S7. Density histogram distributions of vesicles diameter in nonresolving mode S10

Figure S8. Histogram distributions of vesicles in resolving mode S11

Figure S9. Density histogram distributions of vesicles diameter in resolving mode S12

Figure S10. Comparison of the different *m/z* histogram distributions S13

Figure S11. Measurement time series of the vesicle count rate in nonresolving mode S14

Lipid vesicles number calculation S14

Figure S12. Heatmaps for the charge vs. mass for vesicles S15

Figure S13. DLS analysis of the blood serum sample S16

Figure S14. Histogram distributions for serum samples in nonresolving mode S17

Figure S15. Histogram distributions for serum samples in resolving mode S18

References S19

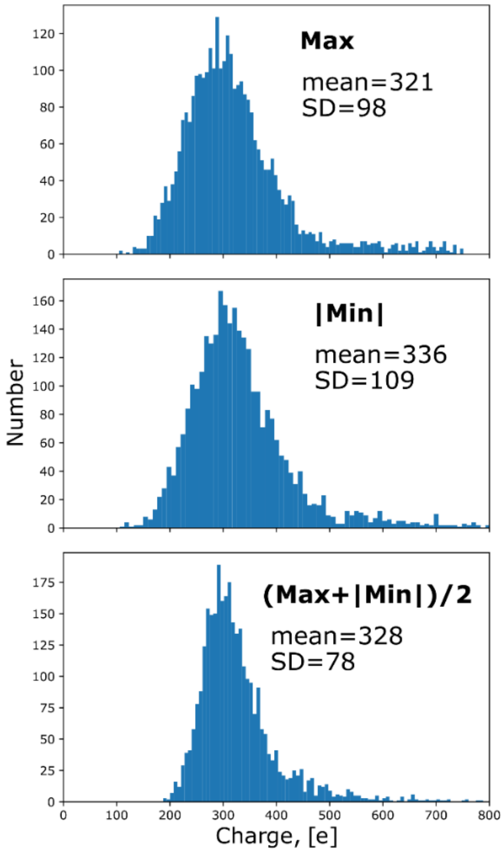

Figure S1. Histogram plots to demonstrate charge measurement error. The range of 0 to 400 us of transients that pass the validation as ‘good’ was used as there should be a blank signal without the ‘entrance’ peak from the charged particle. The maximum, minimum, and average of absolute maximum and minimum values are displayed as 100-bin histograms with their mean and standard deviation values denoted.

Charge detector measurement error

Assuming the mean of blank 328 e as a charge measurement error, it is possible to estimate the most conservative propagation of relative error toward diameter measurement for the lowest charge value 600 e and $RSD_{m/z}$=11.6% of *m/z* 2.5×10^5 measurement (see Fig. S10):

$$\frac{\Delta m}{m} = \frac{\Delta z}{z} + RSD_{m/z} = \frac{328}{600} + 0.116 = 0.66$$

$$\frac{\Delta d}{d} = \frac{1}{3}\frac{\Delta m}{m} = 0.22$$

Table S1 The voltage and frequency values for different *m/z* settings used for UQ and DQ. Note that UQ was used only with 0 V DC in this work.

	UQ			DQ		
m/z	DC, V	AC, V	F, Hz	DC, V	AC, V	F, Hz
2×10^5	15.11	100	13867.72	30.07	199.12	24718.35
2.5×10^5	15.11	100	12403.66	30.07	199.08	22106.65
5×10^5	15.11	100	8770.72	30.06	199.01	15629.09
5.5×10^5	15.11	100	8362.55	30.06	199.01	14901.52
7×10^5	15.11	100	7412.61	30.06	198.99	13208.31
1.2×10^6	15.11	100	5661.47	30.06	198.97	10087.34
2×10^6	15.11	100	4385.36	30.06	198.97	7813.73
3×10^6	15.11	100	3580.63	30.06	198.98	6379.95

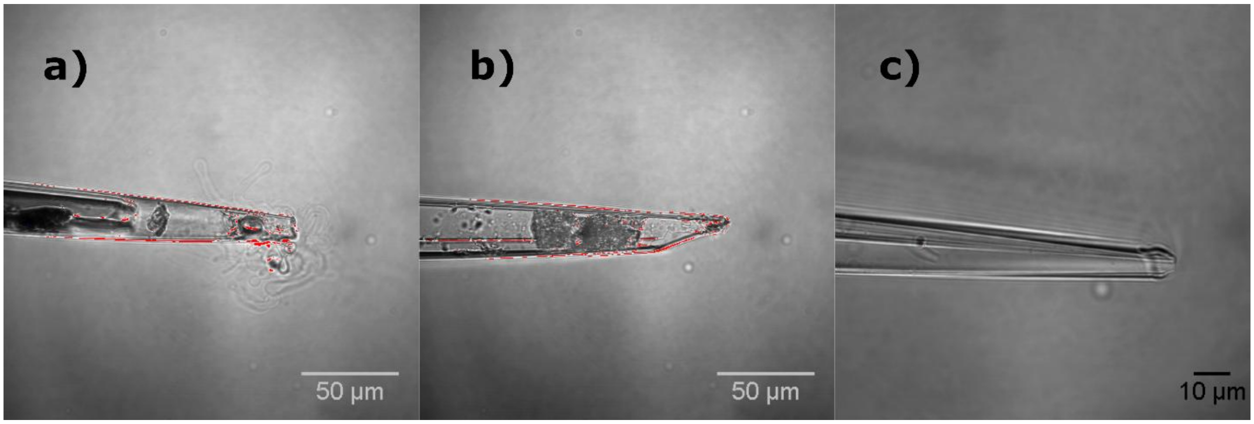

Figure S2. Microphotography of nanoelectrospray emitters taken after their usage: a) Sample 1, b) Sample 2, c) Sample 3. Samples with vesicles in 100 mM ammonium acetate were prepared from the same initial suspension of vesicles. The emitter tip was opened by touching the sampling capillary (SC) surface and then translated away from it. The procedure was repeated until there was no corona discharge on the tip at about 1.5-2 mm between the tip and SC.

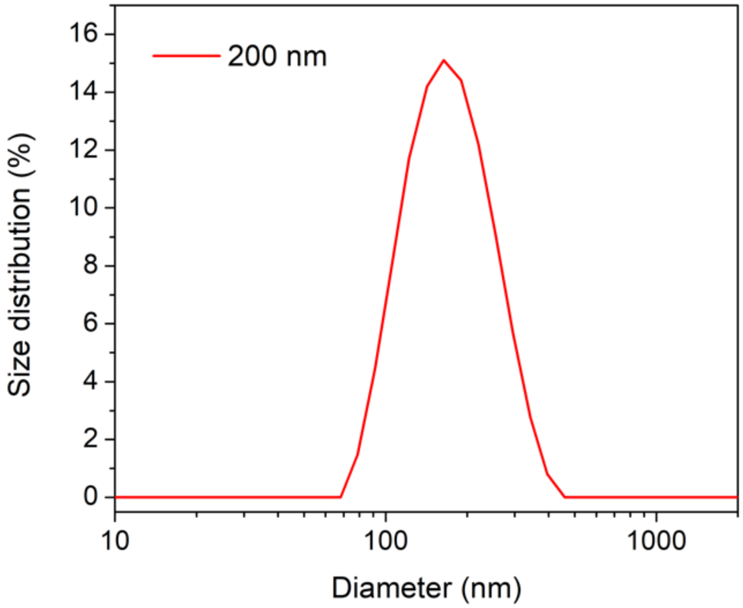

Figure S3. Dynamic light scattering (DLS) analysis of the prepared 200 nm vesicle sample. The sample was diluted to the 0.2 mM lipid concentration, transferred to a plastic disposable cuvette, and measured after 2 minutes of equilibration at 298 K. The z-average value is 159 nm.

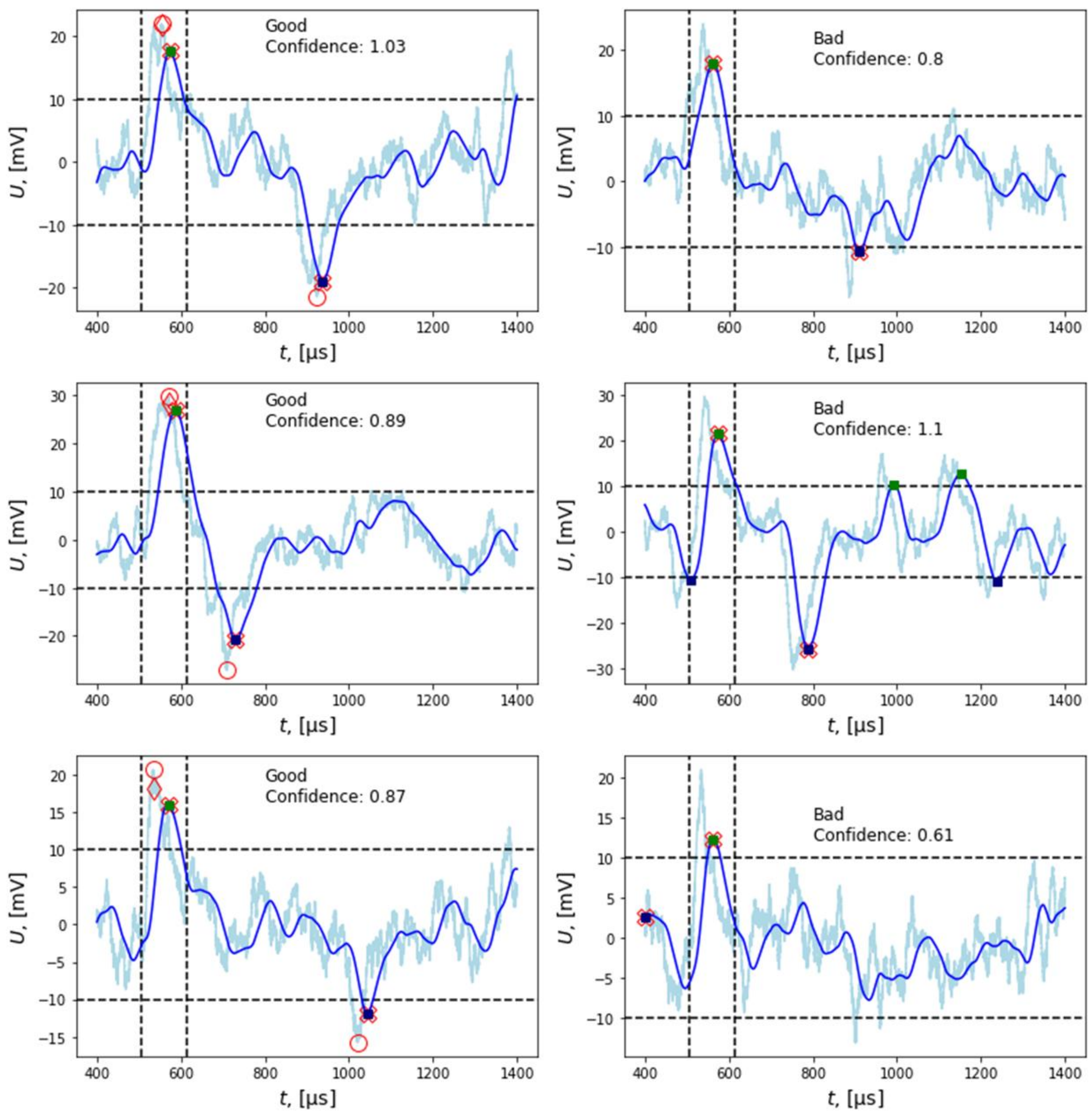

Figure S4. Examples of the charge detector signal postprocessing. Raw data are shown in light blue and filtered data in blue. The signal processing was done using the SciPy python package. The second-order Butterworth filter with 0.1-100 kHz bandpass was utilized. Vertical black dashed lines show the range set for the expected first peak, and horizontal black dashed lines show a threshold of |10| mV for the peak detection. Peaks of the filtered signal are detected with parameters width=50, prominence=10, range |10|-|100| mV. Markers designate detected peaks as follows: maximum and minimum of raw signal (red circles), absolute average between maximum and minimum of raw signal (red diamond) used for charge calculation, detected peaks of filtered signal (green and blue filled squares) and peaks of filtered signal used for *m/z* calculation (red crosses). The confidence is calculated as the ratio $(|U_{max}|+|U_{min}|)/(2U_{max})$.

The confidence value used here is 0.8. Only signals with single positive and single negative peaks and confidence higher than 0.8 are considered ‘good’, all others as ‘bad’. However, this approach is rather conservative, as all transients where two particles were recorded are discarded. In some cases though, signals were falsely attributed as ‘good’ due to noise. Thus, random selections of 100 signals from ‘good’ were additionally visually inspected to estimate the fraction of signals falsely assigned to ‘good’. The summary is listed in Table S1.

Table S2 Samples comparison and summary of the validation

	Day of preparation since original vesicles produced	Emitter tip, µm	Fraction of ‘bad’ signals		Fraction of signals falsely assigned to ‘good’
			Non-resolving	Resolving	
Sample 1	6th	9	31%	14%	7%
Sample 2	8th	16	49%	14%	10%
Sample 3	11th	3	50%	17%	9%

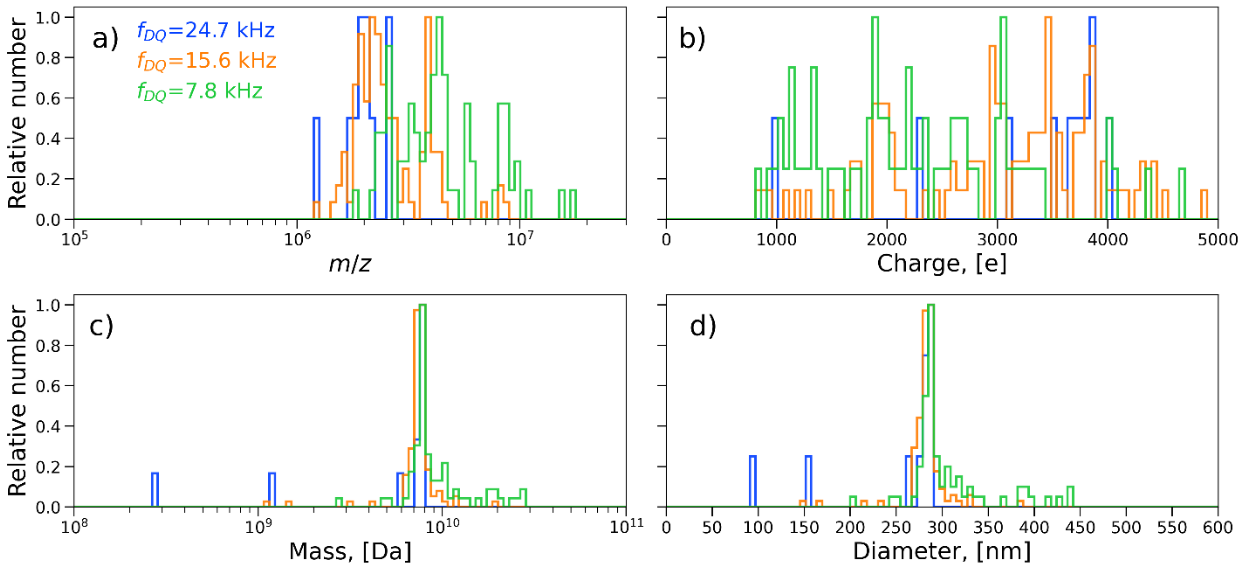

Figure S5. Histogram distributions of *m/z*, charge, mass, and diameter for 300 nm polystyrene beads sample when emitter 2 was used. The bin number in each histogram is 100 with equal widths (b, d) and varying widths spaced evenly on a log scale (a, c).

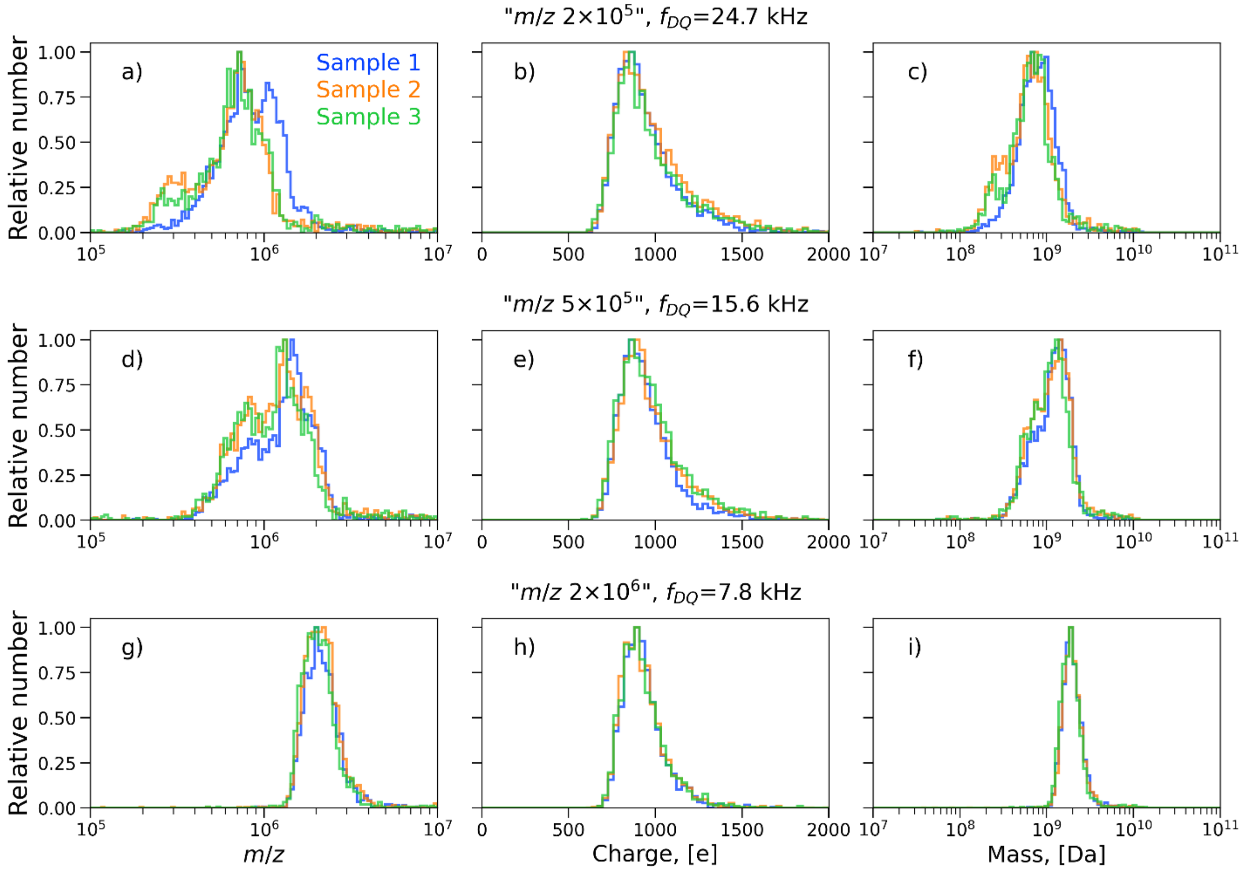

Figure S6. Histogram distributions of *m/z*, charge, and mass as measured by the charge detector at three different nonresolving DQ settings (DC component is 0 V) for three studied samples. The bin number in each histogram is 100, with equal widths for charge and varying widths spaced evenly on a log scale *m/z* and mass.

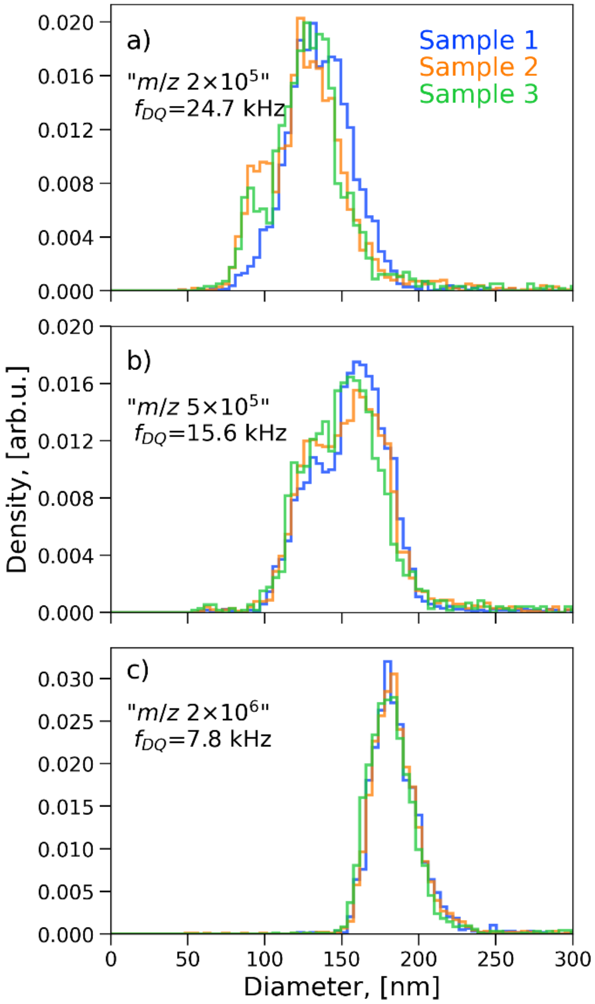

Figure S7. Density histogram distributions of vesicles derived from the mass using the density 997 kg/m^3 for three studied samples at three different quadrupole nonresolving (DC component is 0 V) settings. The bin number in each histogram is 100 with equal widths.

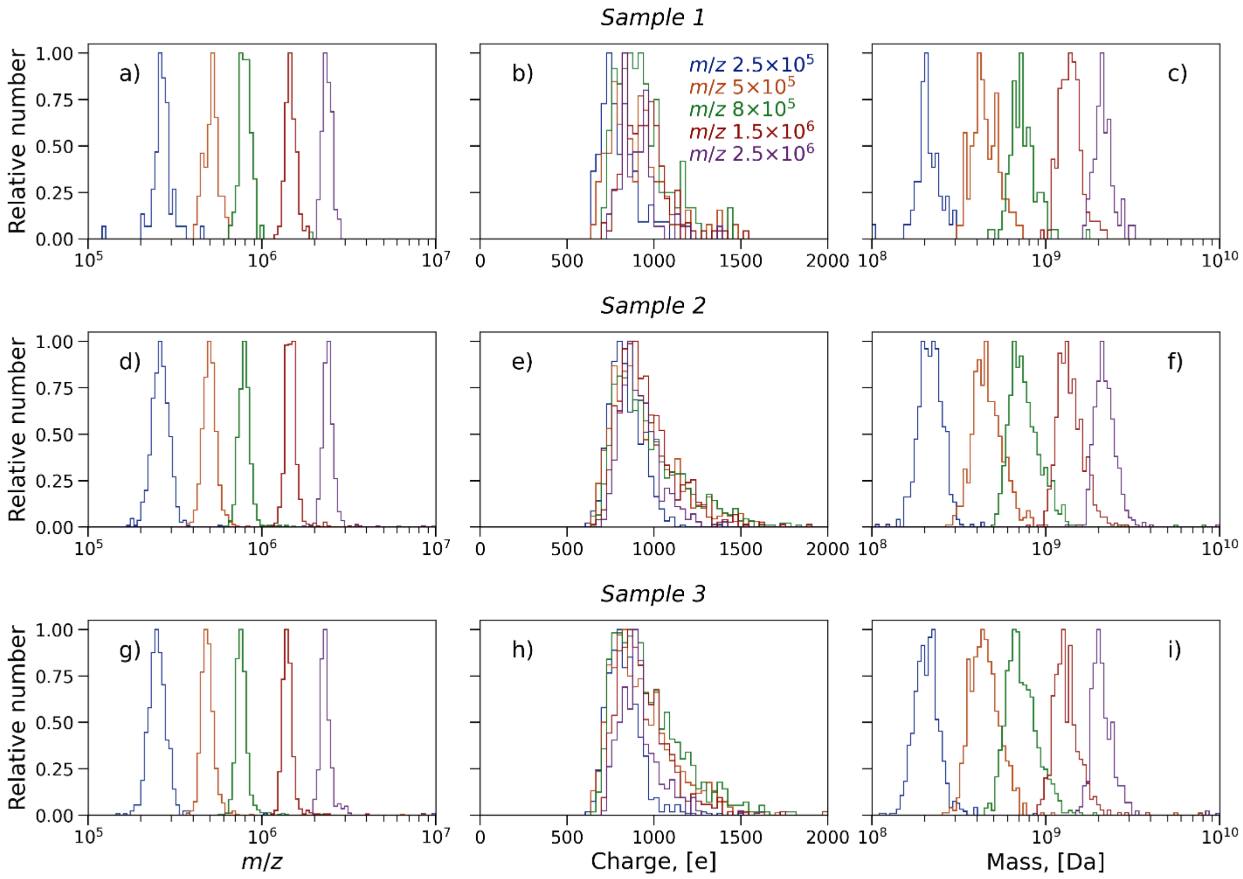

Figure S8. Histogram distributions of *m/z*, charge, and mass as measured by the charge detector at five different *m/z* resolving DQ settings for three studied samples. The bin number in each histogram is 100, with equal widths for charge and varying widths spaced evenly on a log scale for *m/z* and mass.

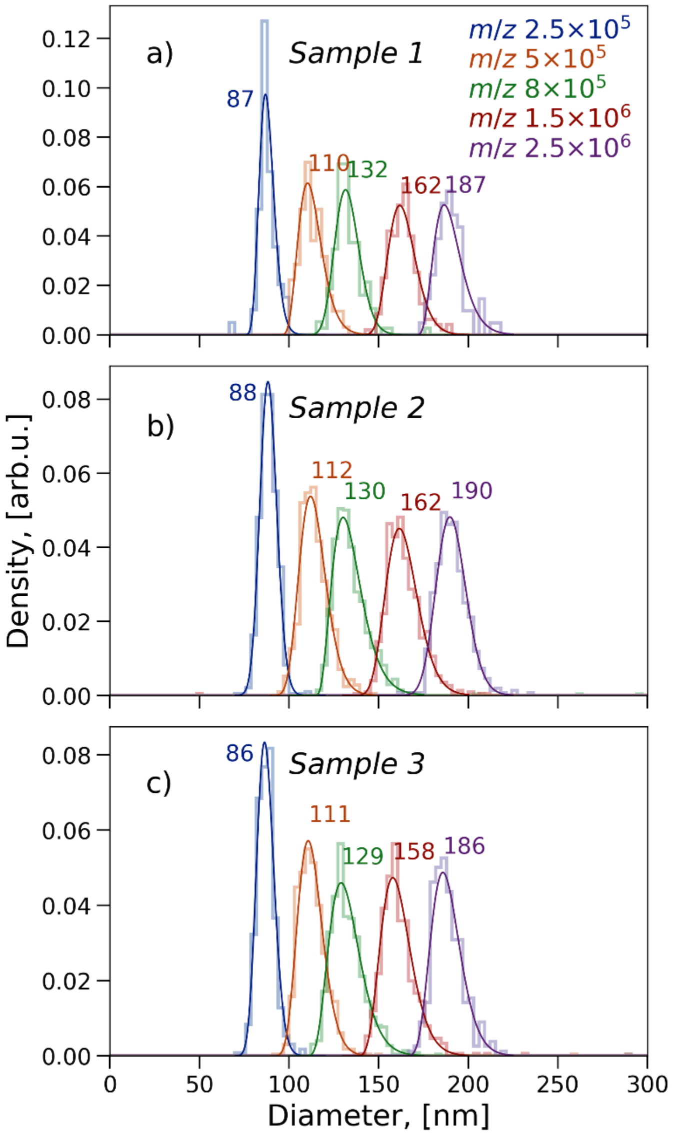

Figure S9. Density histogram distributions of vesicles derived from the mass using the density 997 kg/m^3 at five different *m/z* resolving DQ settings for three studied samples. The modal values of the fitted gamma distributions are shown near the corresponding diameter histogram.

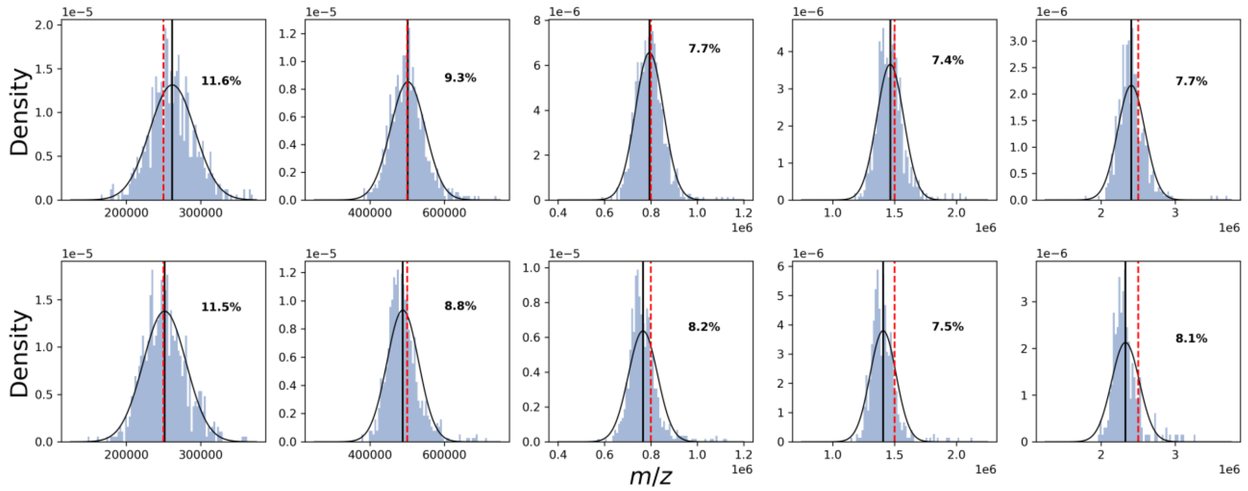

Figure S10. Comparison of the different *m/z* histogram distributions in resolving DQ settings at 90% (top) and 95% (bottom) resolution settings. The resolution percent tune here is the fraction of the maximum theoretical resolution ~0.168, i.e., the ratio between DC and AC components of the harmonic potential applied to quadrupole rods. Practically, it means that DC voltage is lower by 10% or 5% from the maximum theoretical value. The Gaussian distributions are fitted to calculate relative standard deviation values denoted in bold. Black vertical lines represent the mean value of fitted Gaussian distributions, and red dashed lines represent the m/z_{DQ}.

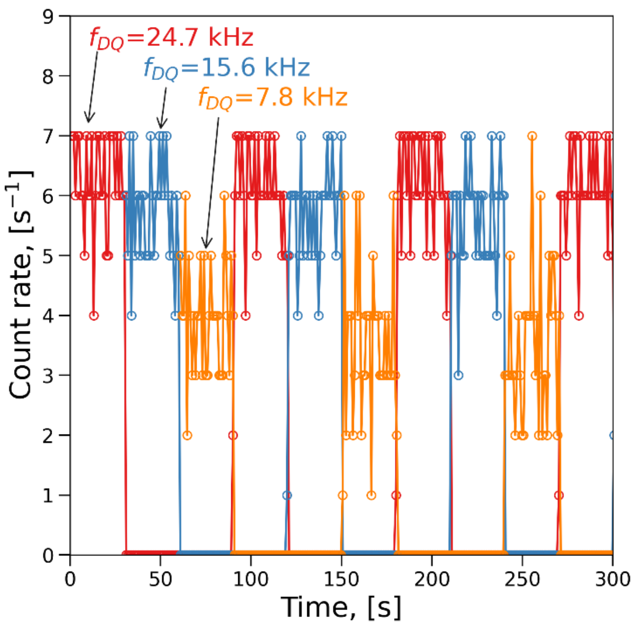

Figure S11. Measurement time series of the vesicle count rate as detected by the charge detector at three frequency settings of DQ in nonresolving mode cycled every 30 s.

Lipid vesicles number calculation

Total number of lipid molecules per vesicle[1]:

$$N_{tot} = \frac{4\pi r^2 + 4\pi(r-h)^2}{a} = 2.1 \times 10^5$$

where r = 160 nm is the vesicle radius, h = 5 nm is the thickness of the phospholipid bilayer, a = 0.71 nm^2 is the phospholipid head group area. Number of vesicles per nL then:

$$N_{ves} = \frac{M \times N_A}{N_{tot} \times 10^9} = 1.4 \times 10^6 \text{ nL}^{-1}$$

where M = 0.5 mM is the molar concentration of the lipids, and N_A = 6.02×10^{23} is the Avogadro number. Taking the flow rate of nano-electrospray as 50 nL/min, the number of vesicles released from the emitter per minute is 7.1×10^7.

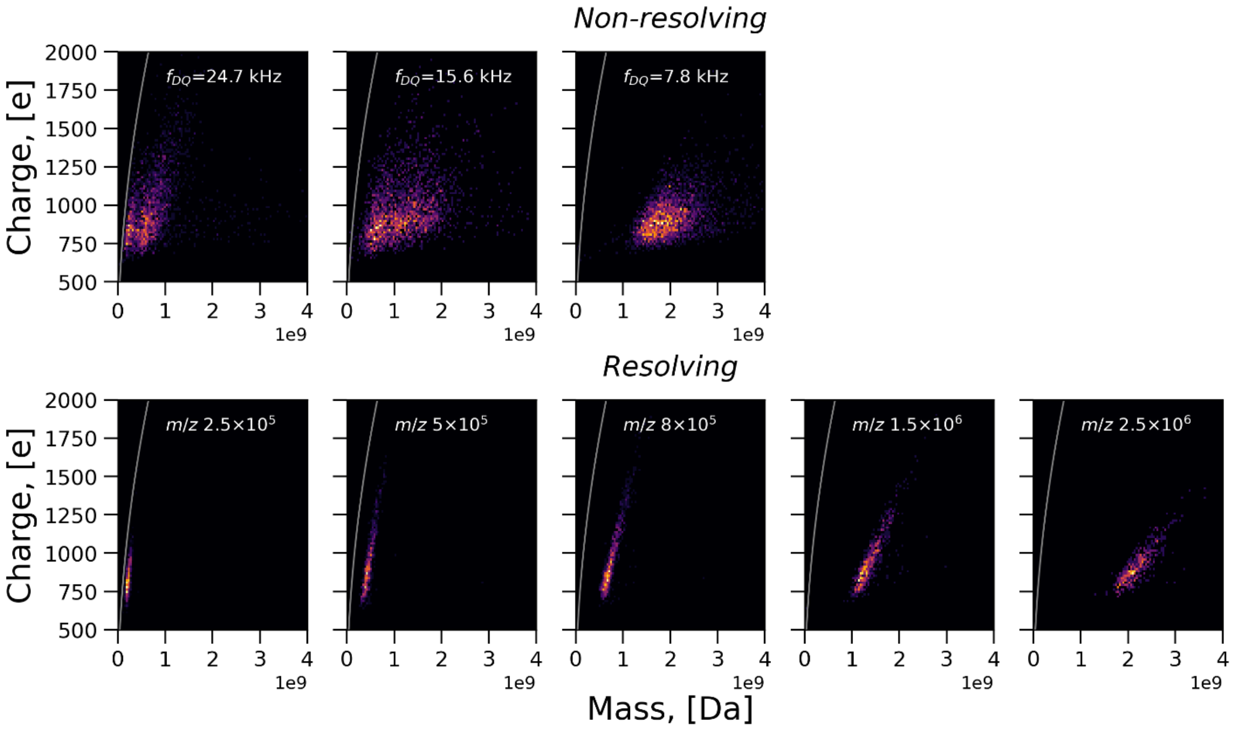

Figure S12. Heatmaps for the charge vs mass of the detected vesicles in the nonresolving and resolving modes. The grey line represents Rayleigh's charge limit for water.

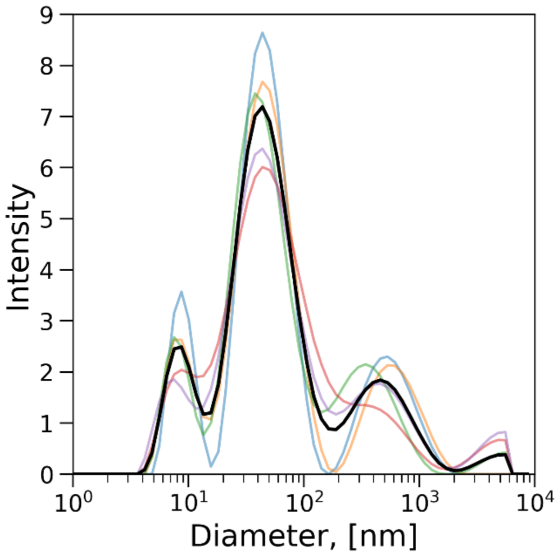

Figure S13. Dynamic light scattering (DLS) analysis of the blood serum sample. The sample was diluted with water at a 1:1 (v/v) ratio, transferred to a plastic disposable cuvette, and measured after 2 minutes of equilibration time at 298 K. The thin colored lines depict five independent measurements, and the bold black line is their average.

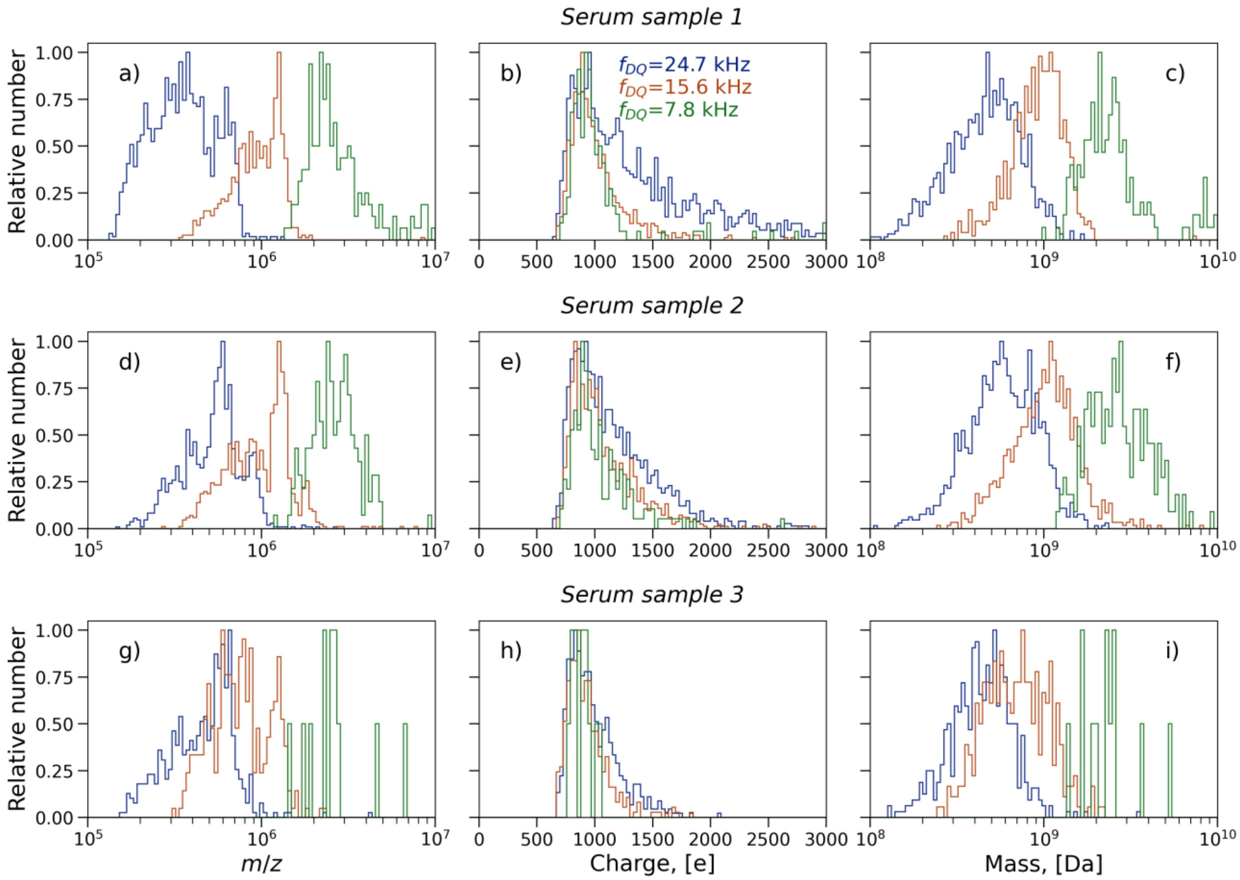

Figure S14. Histogram distributions of *m/z* (first column), charge (second column), and mass (third column) of particles in three serum samples as detected by CD at f_{DQ} 24.7, 15.6, and 7.8 kHz. The bin number in each histogram is 100 with equal widths (b, e, h) and varying widths spaced evenly on a log scale (a, c, d, f, g, i).

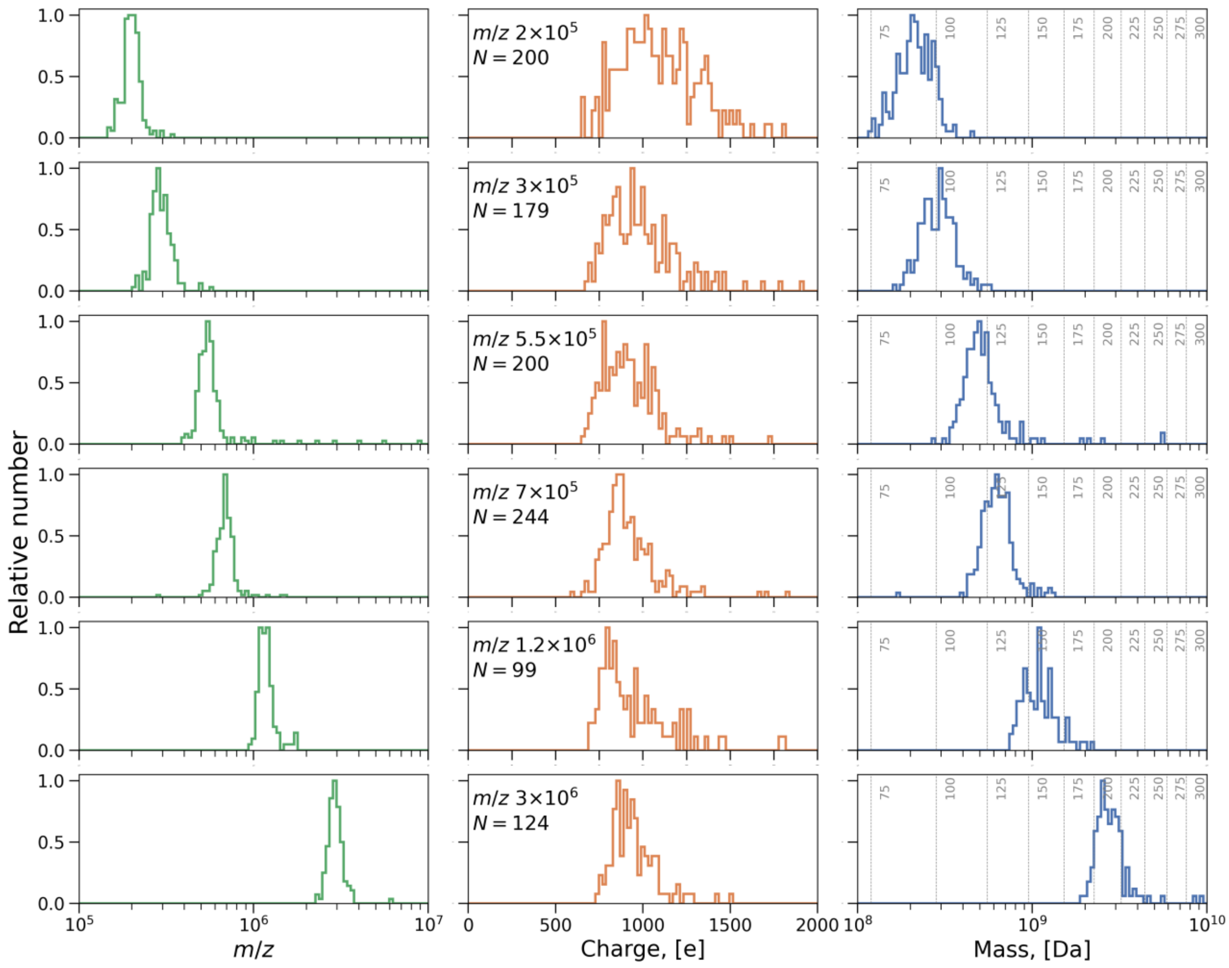

Figure S15. Histogram distributions of *m/z* (first column), charge (second column), and mass (third column) for serum sample particles as detected by CD at m/z_{DQ} 2×10^5, 3×10^5, 5.5×10^5, 7×10^5 1.2×10^6, 3×10^6. In the bottom plot, the grey vertical lines denote mass values equivalent to spherical particles with a density of 997 kg m^{-3} for different diameters in nanometers. The bin number in each histogram is 100, with equal widths for charge and varying widths spaced evenly on a log scale for *m/z* and mass.

References

1. Mozafari, M. R.; Mazaheri, E.; Dormiani, K., Simple Equations Pertaining to the Particle Number and Surface Area of Metallic, Polymeric, Lipidic and Vesicular Nanocarriers. *Sci Pharm* **2021,** *89* (2).
